# Engineering Charge Heterogeneity in COF/Graphene Hydrogels for Salt‐Resistant Solar Evaporation

**DOI:** 10.1002/advs.202519171

**Published:** 2025-12-02

**Authors:** Shuwen Jia, Yanrui Li, Guangyu He, Xinda Nan, Xiao Xiao, Chuan Wang, Arne Thomas, Changxia Li

**Affiliations:** ^1^ School of Chemistry and Molecular Engineering Nanjing Tech University Nanjing 211816 China; ^2^ Department of Chemistry Westlake University Hangzhou Zhejiang 310030 China; ^3^ Department of Chemistry, Division of Functional Materials Technische Universität Berlin Hardenbergstraße 40 10623 Berlin Germany

**Keywords:** covalent organic frameworks, evaporation, heterocharged hydrogel, salt‐resistant

## Abstract

Solar‐driven interfacial evaporation technology offers a sustainable solution for water treatment, however, its efficiency in marine environments is often hindered by salt accumulation. Here, a series of covalent organic framework (COF)/graphene hydrogels with controllable charge distributions is reported, including homo‐ and hetero‐charged as well as zwitterionic surface functionalities. By adjusting the ratio of anionic and cationic building blocks during COF synthesis, the surface charge of the hydrogel evaporator is continuously modulated at the molecular level. The hetero‐charged and zwitterionic hydrogels enable faster water evaporation in seawater than in pure water by reducing the evaporation enthalpy. In particular, the hetero‐charged hydrogel achieves an evaporation rate of up to 3.23 kg m^−2^ h^−1^ in 3.5 wt.% saline water, 12% higher than that in pure water, and maintains strong salt resistance even in 20 wt.% brine. Beyond desalination, the hydrogel exhibits high potential for treating diverse wastewaters, providing a scalable platform for sustainable water treatment in complex environments.

## Graphical abstract

Hetero‐charged COF/graphene hydrogel (HCG‐1) with spatially separated cationic and anionic domains is engineered to achieve salt‐resistant solar evaporation. This unique charge architecture regulates Na⁺ and Cl^−^ distribution, promotes intermediate‐water formation and effectively suppresses salt crystallization, enabling stable solar evaporation in saline water at rates exceeding those in pure water.

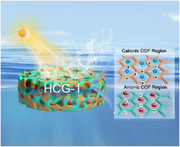

## 1 Introduction

Freshwater scarcity, driven by rapid population growth, climate change, and industrialization, has become a critical global issue, affecting ≈2 billion people.^[^ ^1^, ^2^ ^]^ To address this crisis, seawater desalination technologies have emerged as a major research focus.^[^ ^3^ ^]^ Amongst these technologies, interfacial solar evaporation has attracted major interest owing to its reliance on sustainable solar energy, environmentally friendly nature and often‐excellent water evaporation performance, highlighting its considerable potential for future development.^[^ ^4^, ^5^, ^6^, ^7^ ^]^

The key to the interfacial solar evaporation technique lies in the development of high‐performance photothermal materials capable of efficient solar‐to‐heat conversion. Currently, various types of photothermal materials, including carbon‐based materials,^[^ ^8^, ^9^, ^10^, ^11^, ^12^ ^]^ semiconductors,^[^ ^13^, ^14^ ^]^ polymers,^[^ ^15^, ^16^ ^]^ and metal nanoparticles,^[^ ^17^, ^18^ ^]^ have shown promising evaporation efficiencies.^[^ ^19^ ^]^ However, their application in real seawater scenarios remains limited owing to salt accumulation, material degradation, and high production costs.^[^ ^20^, ^21^ ^]^ Salt crystallization on the evaporator surface not only lowers light absorption and thermal efficiency but also increases maintenance requirements. Moreover, the strong hydration of salt ions raises the evaporation enthalpy, resulting in substantially lower evaporation rates in saline water than in pure water.^[^ ^22^ ^]^

To address these challenges in the solar evaporation of seawater, researchers have explored various strategies, such as constructing ion transport channels,^[^ ^23^, ^24^ ^]^ incorporating Janus structures to separate water transport and evaporation zones,^[^ ^25^, ^26^, ^27^, ^28^ ^]^ and designing selective salt crystallization regions or self‐rotating evaporators.^[^ ^29^, ^30^, ^31^, ^32^ ^]^ While these innovations mitigate salt fouling to some extent, achieving higher evaporation rates in saline water than in pure water remains a rarely accomplished goal. Importantly, zwitterionic hydrogels have emerged as promising candidates for salt‐resistant solar evaporators owing to their strong hydration ability, biocompatibility, and environmental friendliness.^[^ ^33^, ^34^, ^35^, ^36^ ^]^ However, the lack of precise control over charge distribution affects their long‐term stability and evaporation efficiency under high‐salinity environments.

On the other hand, covalent organic frameworks (COFs) offer atomic‐level tunability,^[^ ^37^, ^38^, ^39^, ^40^, ^41^ ^]^ precise pore design,^[^ ^42^, ^43^ ^]^ and high thermal and chemical stability, making them attractive for photothermal and water‐interaction engineering.^[^ ^44^, ^45^, ^46^, ^47^, ^48^ ^]^ However, most COFs are synthesized in a powder form, lacking macroscopic connectivity and processability. The integration of COFs into 3D salt‐resistant evaporator architectures with effective water/ion transport channels remains a substantial challenge.

**Figure 1:**
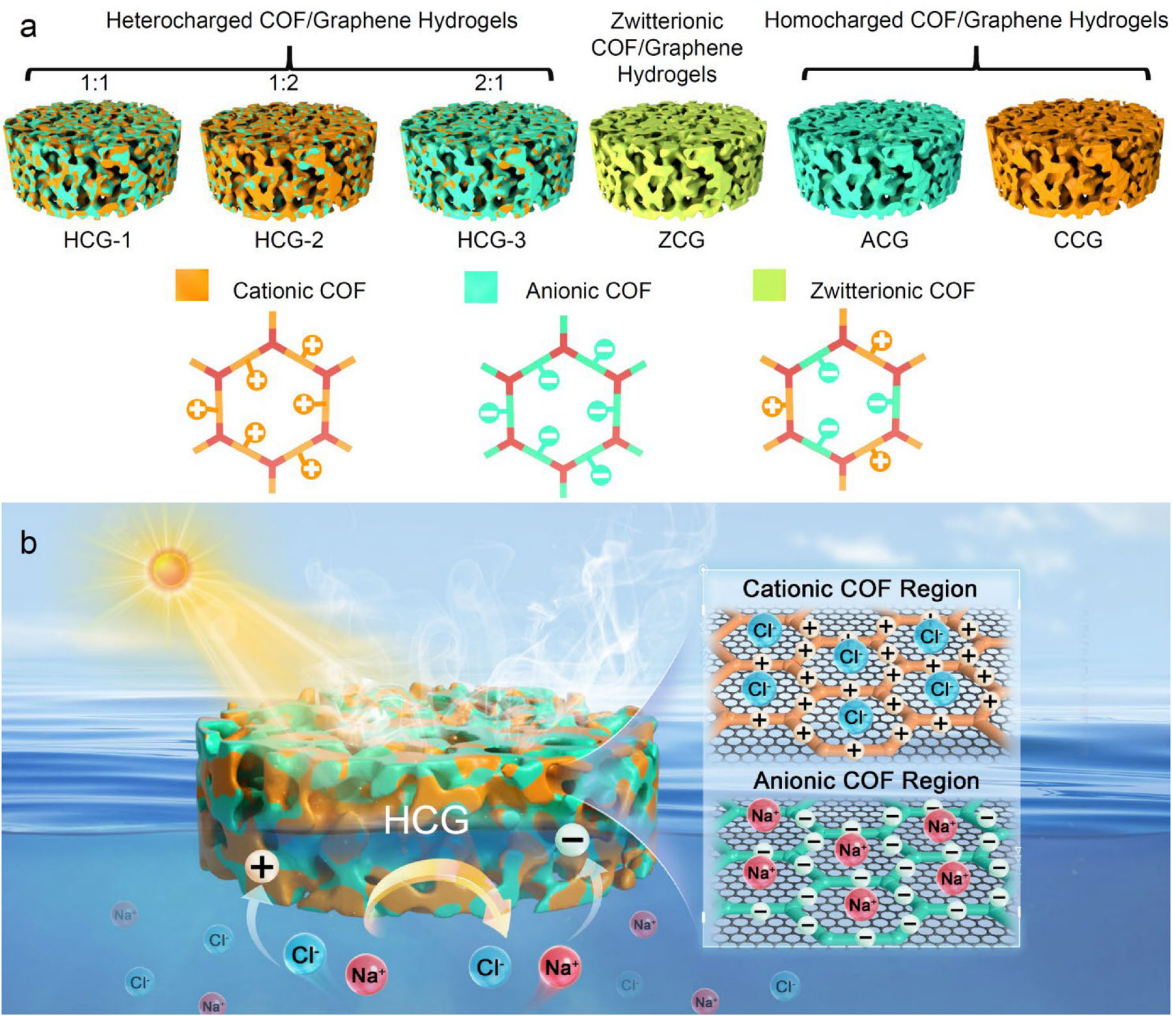
a) Schematic structure of COF/graphene hydrogels (CGs) with different charge types. b) Scheme of a heterogeneously charged COF/graphene hydrogel (HCG) evaporator. The heterogeneously distributed C‐ and A‐COFs within the graphene network facilitate selective ion interactions. This spatial ion regulation enhances IW formation, reduces salt crystallization, and promotes stable, high‐efficiency evaporation in saline environments.

Herein, we report a series of COF/graphene hydrogels (CGs) with a tunable charge composition of the COFs grown on graphene sheets, including heterogeneous, homogeneous, and zwitterionic charge distributions, engineered to enhance solar steam generation in saline water (**Figure** **1**). Amongst them, the heterogeneously charged hydrogel HCG‐1, constructed with a 1:1 ratio of cationic and anionic COFs (C‐ and A‐COFs, respectively), effectively suppresses salt crystallization through spatial ion regulation. Spatially separated regions of C‐ and A‐COFs in the graphene network of HCG‐1 promote selective ionic interactions. This spatial ion modulation enhances the formation of intermediate water (IW), reduces salt crystallization, and promotes stable, efficient evaporation in a salt environment. Consequently, the HCG evaporator achieves a high evaporation rate of 3.23 kg m^−2^ h^−1^ in 3.5 wt.% saline water, 12% higher than that in pure water. Additionally, it shows salt resistance, long‐term durability, and applicability for the purification of wastewater containing diverse impurities.

## 2 Results and Discussion

**Figure 2:**
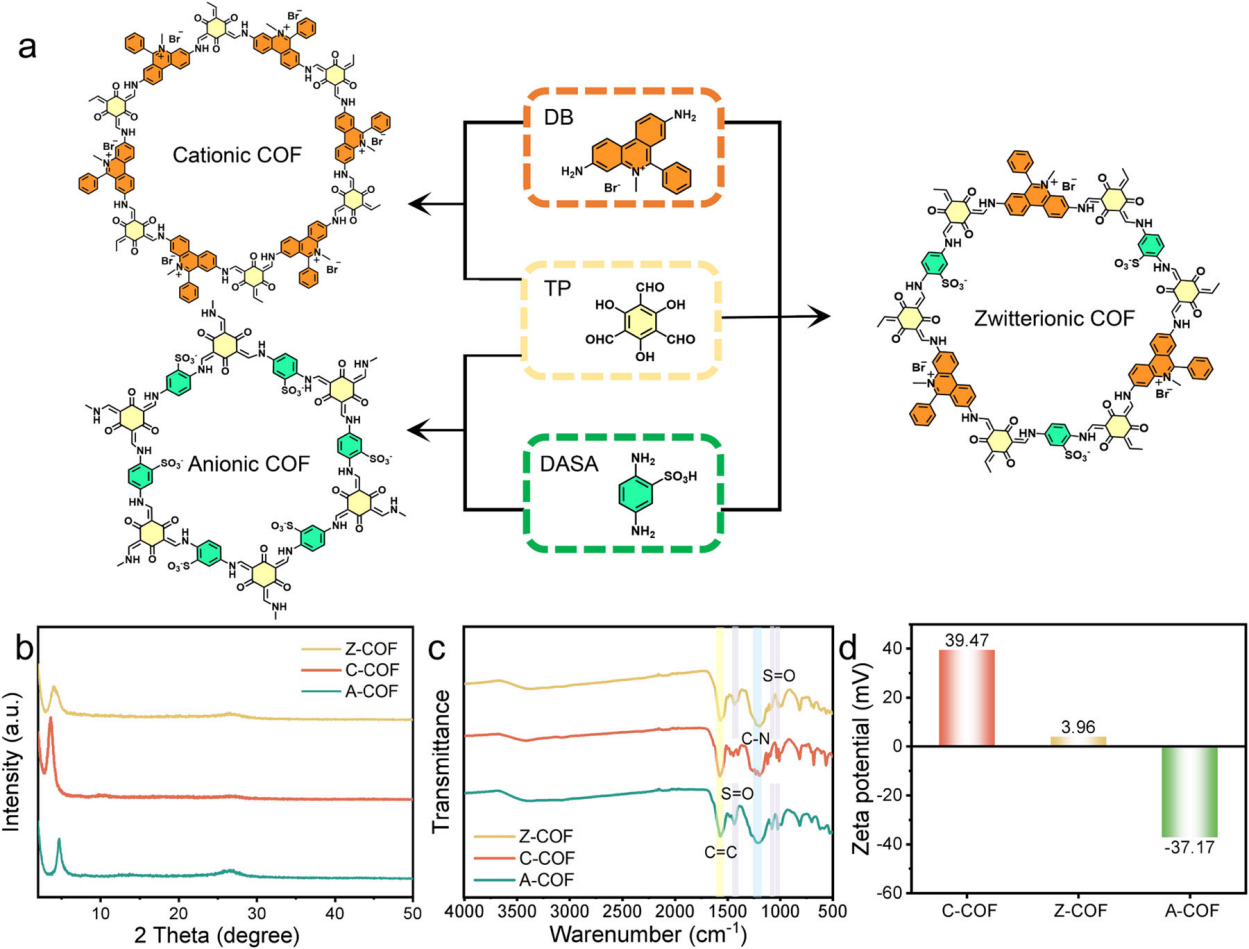
a) Synthetic scheme of C‐COF, A‐COF and Z‐COF. b) XRD patterns, c) FTIR spectra and d) zeta potentials of C‐COF, A‐COF and Z‐COF.

Using a hydrothermal method^[^ ^41^, ^49^, ^50^, ^51^, ^52^ ^]^ with p‐toluenesulfonic acid (PTSA) as the catalyst and water as the sole solvent, we successfully synthesized three types of charged COFs (**Figure** **2**a). Specifically, 1,3,5‐triformylphloroglucinol (Tp), 2,5‐diaminobenzenesulfonic acid (DASA), and dimidium bromide (DB) were used as monomers to prepare a zwitterionic COF (Z‐COF). The mixture was vortexed for 20 min, and the resulting solution was then transferred to an oven and heated at 120 °C, yielding Z‐COF as black powder. Using the same method with the pure anionic or cationic monomers, A‐COF and C‐COF were also synthesized, respectively.

Morphological analysis of the pure COFs via scanning electron microscopy (SEM) revealed distinct structural features. Z‐COF comprised aggregated spherical particles with a diameter of ≈1 µm. Moreover, C‐COF showed a rod‐like morphology, and A‐COF exhibited a cauliflower‐like structure (Figure S1, Supporting Information). The successful synthesis of COFs was confirmed via powder X‐ray diffraction (XRD), Fourier‐transform infrared (FTIR) spectroscopy, and X‐ray photoelectron spectroscopy (XPS) analyses (Figure 2b,c; Figure S2, Supporting Information, respectively). Peaks at 2θ = 3.6°−4.6° and 26.7° in the XRD patterns corresponded to the (100) and (001) crystallographic planes, respectively, indicating a highly ordered COF structure (Figure 2b).^[^ ^41^, ^51^, ^53^ ^]^ The FTIR spectra showed strong characteristic peaks at 1655 cm^−1^ (C = O), 1568 cm^−1^ (C = C) and 1220 cm^−1^ (C–N), which can be attributed to the formation of β‐ketoenamine‐linked frameworks. In addition, the presence of S = O stretching vibrational peaks near 1429, 1077, 1024, and 984 cm^−1^ in A‐COF and Z‐COF further confirmed the presence of the −SO_3_H group (Figure 2c). Zeta potential analysis indicated that C‐COF was positively charged, A‐COF was negatively charged, and Z‐COF was close to neutral (Figure 2d).

N_2_ adsorption–desorption isotherm measurements further revealed that the calculated Brunauer–Emmett–Teller (BET) surface area values for Z‐COF, A‐COF, and C‐COF were 220, 200, and 170 m^2^ g^−1^, respectively, with corresponding pore sizes of 1.70, 1.42, and 1.40 nm (Figure S3, Supporting Information). The specific surface area of A‐COF was comparable to that of A‐COF synthesized via traditional solvothermal methods (158.6−215 m^2^ g^−1^),^[^ ^54^, ^55^ ^]^ indicating that hydrothermal synthesis is effective for preparing A‐COF. However, the BET surface area of hydrothermally synthesized C‐COF was slightly lower than that obtained using the conventional solvothermal method (495 m^2^ g^−1^)^[^ ^56^ ^]^ or a microwave‐assisted solvothermal method (747 m^2^ g^−1^).^[^ ^57^ ^]^ This discrepancy may arise from reduced crystallinity and pore ordering owing to the high polarity of water and limited reaction kinetics.

**Figure 3:**
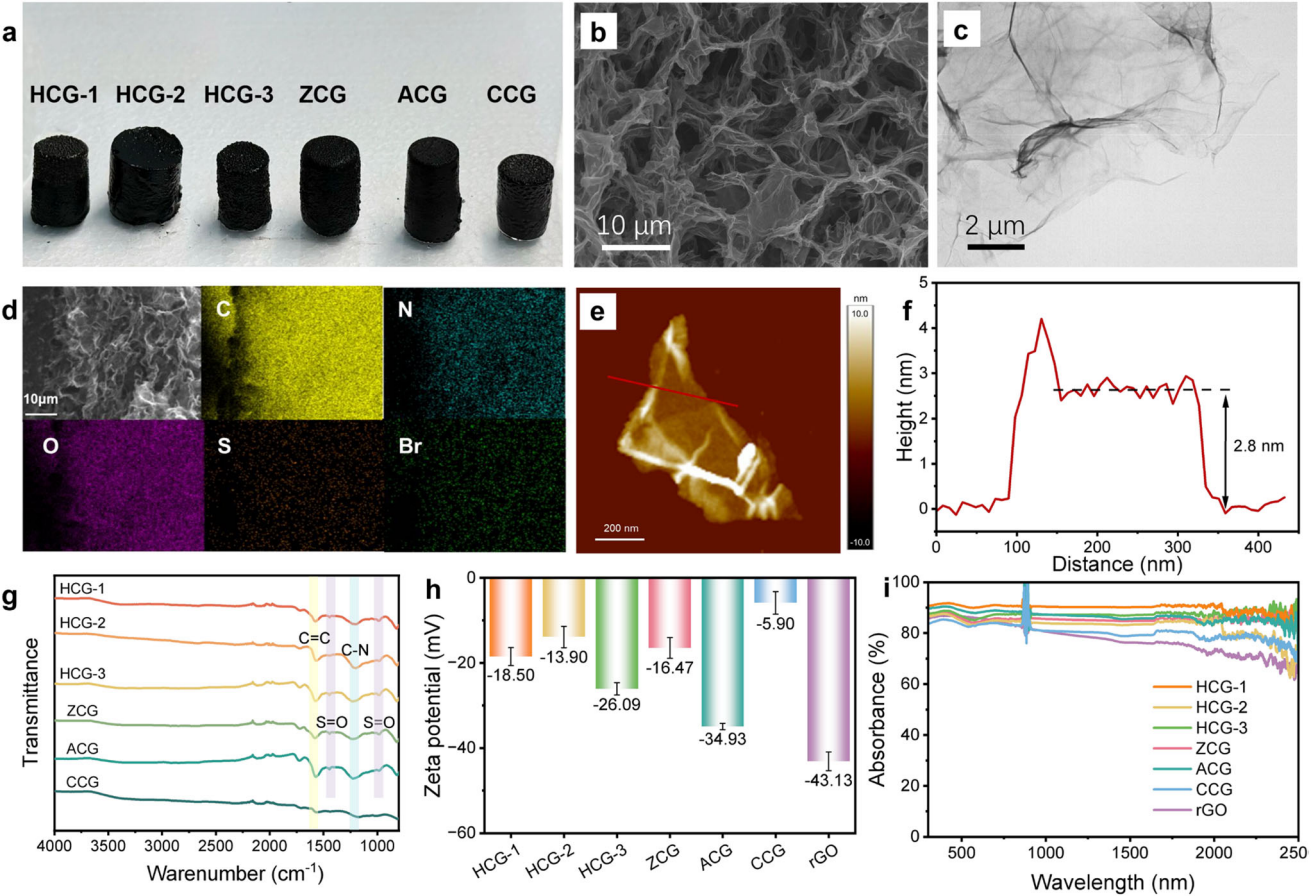
a) Photograph of COF/graphene hydrogels. b) SEM and c) TEM images of HCG‐1. d) SEM image and the corresponding EDS mapping of HCG‐1. e,f) AFM image and the corresponding height profiles of HCG‐1. g) FTIR spectra, h) zeta potentials and i) UV–vis‐NIR spectra of CGs.

Although COFs exhibit excellent chemical tunability and porosity, their powder form lacks the interconnected pore network required for efficient water transport in evaporation systems. To overcome this limitation, we developed CGs by incorporating COF monomers into a graphene oxide (GO) dispersion. The COF monomers were initially mixed with GO via mechanical shaking to form a preliminary composite, followed by additional GO addition to enhance crosslinking within the network. The hydrothermal reaction was performed at 120 °C for the in situ growth of COF on the GO surface while the GO is reduced.^[^ ^50^ ^]^ Upon completion of the reaction and cooling to room temperature, black hydrogels were obtained (**Figure** **3**a).

To systematically study the influence of surface charge on water evaporation performance, hetero‐charged COF/graphene hydrogels (HCG‐1−3), a zwitterionic COF/graphene hydrogel (ZCG), an A‐COF/graphene hydrogel (ACG), and a cationic COF/graphene hydrogel (CCG) were synthesized by introducing different COF monomers during the synthesis (Figure 1a; details are provided in Table S1, Supporting Information). The difference between HCG and ZCG syntheses lies in the order in which the monomers and GO are mixed. For ZCG synthesis, the anionic and cationic monomers are directly combined with GO and Tp and reacted together. However, for HCG synthesis, two separate mixtures are first prepared: one containing the cationic monomer, Tp and GO, and the other containing the anionic monomer, Tp and GO. After a certain period, these mixtures are combined and subjected to hydrothermal treatment. Thus, whereas in ZCG the anionic and cationic units are mixed at the molecular level, in HCG the C‐ and A‐COF patches remain separated at the nanoscale level. Amongst them, the HCGs are denoted as HCG‐1, HCG‐2, and HCG‐3 based on the A‐COF:C‐COF molar ratios, which are 1:1, 1:2, and 2:1, respectively. All hydrogels can be prepared on a large scale by simply using larger autoclaves (Figure S4, Supporting Information).

SEM measurements of freeze‐dried CGs revealed uniformly distributed pore structures with good permeability (Figure 3b; Figure S5, Supporting Information). Representative transmission electron microscopy (TEM) and SEM images showed a rough surface with numerous wrinkles (Figure 3c; Figures S5 and S6, Supporting Information). This structure facilitates water transport by providing continuous evaporation channels, thereby enhancing evaporation efficiency. Notably, no discrete COF rods or particles were observed in any of the CG samples, indicating that the two‐dimensional COFs were formed in situ on the surface of graphene nanosheets. Importantly, although the macroscopic morphology changes significantly owing to the templating effect of graphene, the intrinsic microporous structure of the COFs remains essentially unchanged. Meanwhile, the composites exhibit additional mesopores and macropores originating from the interconnected graphene network, thereby forming a hierarchical porous structure.^[^ ^50^ ^]^ Energy‐dispersive X‐ray spectroscopy (EDS) mapping images showed a homogeneous distribution of C, O, N, S, and Br, indicating that the graphene sheets were uniformly covered by the COF layer (Figure 3d). To further assess COF deposition on graphene, atomic force microscopy (AFM) was used to measure the thickness of the composite nanosheets. A pure reduced graphene oxide hydrogel (rGO) was prepared for comparison. For CGs, the average thickness of the COF‐loaded graphene was 2.6‒4.6 nm (Figure 3e; Figure S7, Supporting Information), compared to ≈1.5 nm for pristine rGO (Figure S7, Supporting Information), confirming the uniform growth of COFs on the graphene surface.

FTIR spectroscopy was used to characterize the chemical composition of CGs. Characteristic peaks at 1578 cm^−1^ (C = C), 1220 cm^−1^ (C–N) and 1442 cm^−1^ and 987 cm^−1^ (S = O stretching vibrations) confirmed the successful formation of COFs on the graphene framework (Figure 3g). Zeta potential measurements were performed to evaluate the surface charge characteristics of CGs (Figure 3h). All samples exhibited negative surface potentials owing to the intrinsic electronegativity of rGO (−43.13 mV). However, the magnitude of the potential varied substantially based on the type and ratio of charged COFs incorporated. HCG‐1 (−18.50 mV) and ZCG (−16.47 mV) exhibited moderate negative potentials, indicating a balanced charge environment resulting from the introduction of A‐COF and C‐COF building blocks with a molar ratio of 1:1. HCG‐2 showed a lower negative value (−13.90 mV), consistent with its higher loading of positively charged C‐COFs, which partially neutralized the negative charge of rGO. In contrast, HCG‐3 (−26.09 mV), with a higher ratio of negatively charged A‐COFs, displayed a higher negative potential than HCG‐1. ACG, composed solely of A‐COFs, exhibited a highly negative potential (−34.93 mV); however, this potential was still less negative than that of pure rGO, reflecting the relatively low surface charge density of A‐COFs compared with that of graphene. The CCG sample, containing only positively charged C‐COFs, had the lowest negative value (−5.90 mV). These results demonstrate that the surface potential of the CGs can be finely tuned by adjusting the charge composition of COFs, validating the molecular‐level controllability of charge distribution in these hydrogels.

The introduction of COFs not only modulates the zeta potential of the hydrogels but also significantly enhances their light absorption properties. As shown in Figure 3i and Figure S8 (Supporting Information), incorporating COFs improves the optical absorption of the hydrogels across a broad wavelength range. In particular, HCG‐1 exhibits outstanding broadband light‐harvesting capability, with an average absorption efficiency of up to 90%. This enhanced light absorption is beneficial for effective photothermal conversion, providing sufficient energy for solar evaporation, even under high‐salinity conditions.

Surface wettability measurements (Figure S9, Supporting Information) revealed water contact angles of 93.2°, 74.3°, 102.8° and 107.4° for HCG‐1, HCG‐2, HCG‐3 and ZCG, respectively, compared to 130.6° for pristine rGO.^[^ ^58^ ^]^ These results indicate that introducing COFs and tuning their proportions can effectively modulate the surface wettability of the hydrogels. Water content tests were conducted to evaluate the hydration capacity of the CGs (Figure S10a, Supporting Information). The water content (*Q*) was calculated using the following formula:

(1) $$Q=M_{w}/M_{d}$$

where *M_w_* and *M_d_* represent the weight of water in the hydrogel and the weight of the corresponding dry aerogel, respectively. All CGs exhibited a significantly higher water content than the pure rGO hydrogel (60 g g^−1^), highlighting the positive effect of COF incorporation on water retention. Specifically, CCG (124 g g^−1^) and HCGs (104‒120 g g^−1^) outperformed ZCG (97 g g^−1^) and ACG (94 g g^−1^), underscoring the advantage of cationic and hetero‐charged frameworks. To further investigate the water transport behavior within the hydrogel network, transport rates were measured (Figure S10b, Supporting Information). Consistent with the trend in water content, all CGs exhibited substantially faster transport than rGO hydrogel (4.04 g min^−1^), with rates of 9.31, 9.06, 8.75, 7.85, 7.28, and 7.07 g min^−1^ for CCG, HCG‐2, HCG‐1, HCG‐3, ZCG, and ACG, respectively. These results clearly suggest that charge composition governs water uptake and transport behavior, with cationic or hetero‐charged configurations favoring enhanced hydration. The observed differences in water content are critical because they directly impact the hydrogel's ability to maintain a continuous water supply to the evaporation interface. A well‐balanced water content facilitates efficient evaporation, while excessively low or high levels can impair performance due to either insufficient replenishment or pore blockage.^[^ ^58^ ^]^ Rapid water transport is thus indispensable for sustaining a stable and efficient evaporation interface, ultimately enabling robust solar‐driven vapor generation.

**Figure 4:**
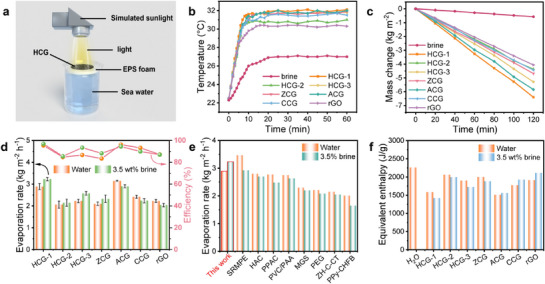
a) Scheme of the apparatus used in the water evaporation experiments. b,c) Time‐dependent temperature and mass change of 3.5 wt.% brine and CGs under 1 sun irradiation (1 kW m^−2^). d) Water evaporation rate and energy efficiency of CGs under 1 sun. The error bars represent standard deviation obtained from five measurements using different samples. e) Evaporation rate comparison of HCG‐1 in water and 3.5% brine with materials from previous studies.^[^ ^32^, ^36^, ^59^, ^60^, ^61^, ^62^, ^63^, ^64^, ^65^ ^]^ f) The evaporation enthalpy of CGs measured via DSC.

The circular CGs (2.1−2.5 cm^2^ area, 2 mm thickness) were placed at the center of an expanded polystyrene (EPS) foam, ensuring that the bottom of the hydrogel directly contacts the water below (**Figure** **4**a). The EPS foam served as a support for holding the hydrogel within the area of simulated vertical solar irradiation, enabling reliable evaluation of the solar steam generation performance over time. Under one‐sun illumination (1 kW m^−2^), the temperature of HCG‐1 increased rapidly and reached a steady state of ≈32 °C in the 3.5 wt.% brine system, while the temperature of brine only increased to ≈27 °C (Figure 4b). After the temperatures stabilized, the change in the water quantity of each sample under identical light conditions was recorded to assess vapor generation performance. The experimental results showed that the water evaporation rate of all CG samples in the 3.5 wt.% brine system was significantly higher than that of pure brine (Figure 4c). In the pure water system, the CGs also demonstrated enhanced photothermal conversion efficiency and evaporation performance (Figure S11, Supporting Information), indicating that the hydrogels have excellent photothermal conversion capability and evaporation performance across different water quality conditions.

The evaporation rate was calculated from the slope of the mass change curve. Notably, the evaporation behavior of the CGs varied depending on the nature and distribution of charges. Hetero‐charged and zwitterionic hydrogels (HCGs and ZCG) exhibited significantly higher evaporation rates in saline water than in pure water (Figure 4d). In contrast, the homo‐charged hydrogels (ACG, CCG and rGO) show lower evaporation rates in saline water. Specifically, HCG‐1 exhibited an evaporation rate of 2.89 kg m^−2^ h^−1^ in pure water, which increased to 3.23 kg m^−2^ h^−1^ in 3.5 wt.% saline water, corresponding to an enhancement of ≈12%. The solar‐to‐thermal conversion efficiency (*η*) was calculated using the following formula:

(2) $$\eta =m\;E_{\mathrm{equ}}/P_{0}$$

Here, *m* represents the net evaporation rate, calculated by subtracting the evaporation rate under dark conditions from the evaporation rate under one‐sun illumination (Figure S12, Supporting Information). *E_equ_* represents the equivalent evaporation enthalpy of water per unit of CGs and *P_0_* represents the solar irradiation power of one sun (1 kW m^−2^). The energy efficiency of HCG‐1 was calculated as 96.9% (Figure 4d), indicating that it is an ideal photothermal material. Most studies report that the evaporation rate of photothermal materials is usually lower in brine than in pure water, primarily owing to pore clogging and solute accumulation during saline evaporation (Figure 4e).^[^ ^32^, ^36^, ^59^, ^60^, ^61^, ^62^, ^63^, ^64^, ^65^ ^]^ In contrast, HCG‐1 developed in this study exhibits higher evaporation rates in salt water than in pure water.

To investigate the underlying mechanism of the enhanced evaporation rates, we measured the evaporation enthalpy (energy consumption for water vaporization) of different samples in both pure water and 3.5 wt.% saline water via dark evaporation experiments and differential scanning calorimetry (DSC) (Figure 4f; Figures S13 and S14, Supporting Information). Both methods consistently demonstrate that heterogeneously charged and zwitterionic hydrogels (HCGs and ZCG) exhibit lower evaporation enthalpies in saline water than in pure water. In contrast, homocharged hydrogels (ACG, CCG, and rGO) show the opposite trend. For instance, HCG‐1 displays a DSC‐measured equivalent evaporation enthalpy of 1419 J g^−1^ in saline water, which is lower than its value of 1583 J g^−1^ in pure water, indicating that less energy is needed for the liquid‐to‐vapor phase transition in saline water.

**Figure 5:**
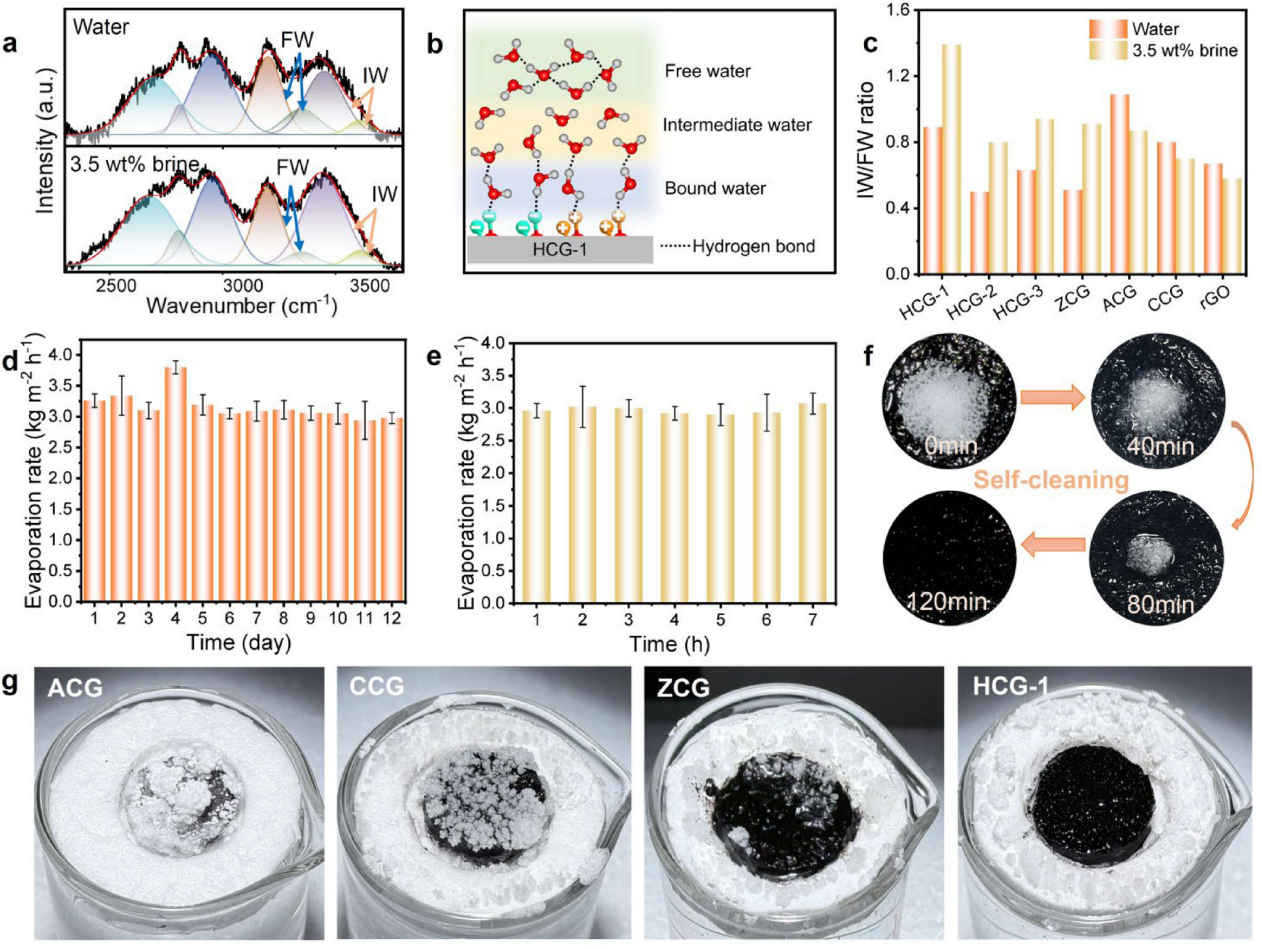
a) Raman profile of HCG‐1 in water and 3.5 wt.% brine. b) Schematic illustration of water molecule types in the hydrogel network. c) The ratio of intermediate water (IW) and free water (FW) in CGs. d) Stability test applying 3.5 wt.% brine and HCG‐1 over 12 days. e) Evaporation rate of HCG‐1 in real seawater under 1 sun. f) Photographs of HCG‐1 with salt crystals on the surface over time. g) Photographs of ACG, CCG, ZCG, and HCG‐1 after 12 h continuous evaporation tests with 20 wt.% brine under 1 solar radiation.

To elucidate the water states within the hydrogels, Raman spectroscopy was employed to investigate the O‒H stretching region of the CGs (**Figure** **5**a; Figures S15 and S16, Supporting Information). Water confined in hydrogels can be categorized into three states: bound water (BW), intermediate water (IW) and free water (FW) (Figure 5b).^[^ ^5^ ^]^ In the spectra, the three peaks at low wavenumber represent vibration of chemical bonds (2600–3000 cm^−1^) and Fermi resonance (3060 cm^−1^).^[^ ^32^, ^59^ ^]^ Peaks at 3200 and 3350 cm^−1^ were assigned to FW, which forms a full network of hydrogen bonds, whereas peaks at 3450 and 3610 cm^−1^ correspond to IW, which engages in weaker or disrupted hydrogen bonds with its surroundings. Notably, IW molecules are more activated than FW, requiring less energy to evaporate and thus being crucial for efficient solar‐driven evaporation.^[^ ^5^, ^15^ ^]^ Raman spectroscopy curve fitting analysis indicates that HCGs and ZCG exhibit significantly higher IW content in saline water than in pure water (Figure 5c). Under normal conditions, brine typically shows a higher evaporation enthalpy than pure water because Na^+^ and Cl^−^ strongly interact with surrounding water molecules through hydration shells, stabilizing them and hindering their evaporation.^[^ ^21^, ^66^ ^]^ However, the situation is reversed in our hetero‐charged and zwitterionic hydrogels. The quaternary ammonium cations in C‐COF disrupt the hydrogen‐bonding network of water and electrostatically bind Cl^−^ from brine, while the sulfonate anions in A‐COF interact with Na^+^ and reduce their stabilizing effect on surrounding water molecules. By immobilizing Na^+^ and Cl^−^ within the hydrogel network, these ionic domains suppress the formation of rigid hydration shells in bulk brine and promote the generation of IW, which is less strongly hydrogen‐bonded and requires lower energy for evaporation. These effects are most pronounced in HCG‐1, which achieves the highest IW/FW ratio of 1.39 in saline water. This can be attributed to its heterogeneously distributed cationic and anionic domains, which spatially separate Na^+^ and Cl^−^ within the hydrogel network, reducing ion clustering and maintaining a stable hydration environment conducive to IW formation. This unique charge architecture not only enhances salt rejection but also creates a dynamic microenvironment that supports rapid water evaporation even under high salinity.

The operational lifetime of solar evaporators is critical for practical desalination. Remarkably, HCG‐1 maintained a stable evaporation rate in a 3.5 wt.% NaCl solution over 12 days, indicating its excellent durability and potential for long‐term solar desalination applications (Figure 5d). Throughout this process, HCG‐1 retained its structural stability and hydrophilicity, as shown by the FTIR and water transport rate measurements (Figure S17, Supporting Information). Its practical utility was further demonstrated using real seawater from the Yellow Sea under one‐sun illumination, achieving an average evaporation rate of 2.97 kg m^−2^ h^−1^—slightly higher than that in pure water—while maintaining stable, high‐efficiency seawater evaporation over 7 h of continuous operation, with no visible salt precipitation on the evaporator surface (Figure 5e; Figure S18, Supporting Information). These results underscore HCG‐1′s considerable potential for practical application in solar‐powered water purification systems and natural marine environments. To further evaluate salt tolerance, HCG‐1 was tested at varying salinities (0, 3.5, 7, 10 and 20 wt%), yielding evaporation rates of 2.89, 3.23, 2.51, 2.30, and 2.06 kg m^−2^ h^−1^, respectively (Figure S19, Supporting Information). These results confirm that HCG‐1 maintains high evaporation efficiency in saline environments and exhibits sensitivity to salinity levels. In addition, 0.2 g of salt crystals (NaCl) were deliberately deposited onto the surface of HCG‐1, which was placed on a 3.5 wt.% saline solution. Remarkably, the salt dissolved gradually and disappeared completely within 120 min, leaving no residual, demonstrating the self‐cleaning ability of HCG‐1 (Figure 5f).

To further elucidate the anti‐salt‐fouling capability of HCG‐1, we investigated the evaporation behavior of various hydrogels in highly concentrated saline (20 wt.% NaCl) under continuous solar irradiation for 12 h (Figure 5g). Striking differences in salt deposition were observed across the hydrogel surfaces. Homo‐charged COF‐based hydrogels (CCG and ACG) suffered from severe salt fouling. The surface of ACG was nearly completely covered by a thick salt crust, severely blocking the evaporation interface, while that of CCG displayed relatively milder crystallization, with parts of the hydrogel surface remaining visible and some salt crystals transferred onto the surrounding foam. In contrast, ZCG exhibited considerably lighter salt deposition, with only a few crystals observed on the hydrogel surface and most salts formed on the foam support. Strikingly, HCG‐1 showed a sharp contrast to all the above systems as its surface remained completely free of visible salt deposition and the evaporation interface stayed consistently clean and continuous, demonstrating excellent resistance to salt crystallization.

**Figure 6:**
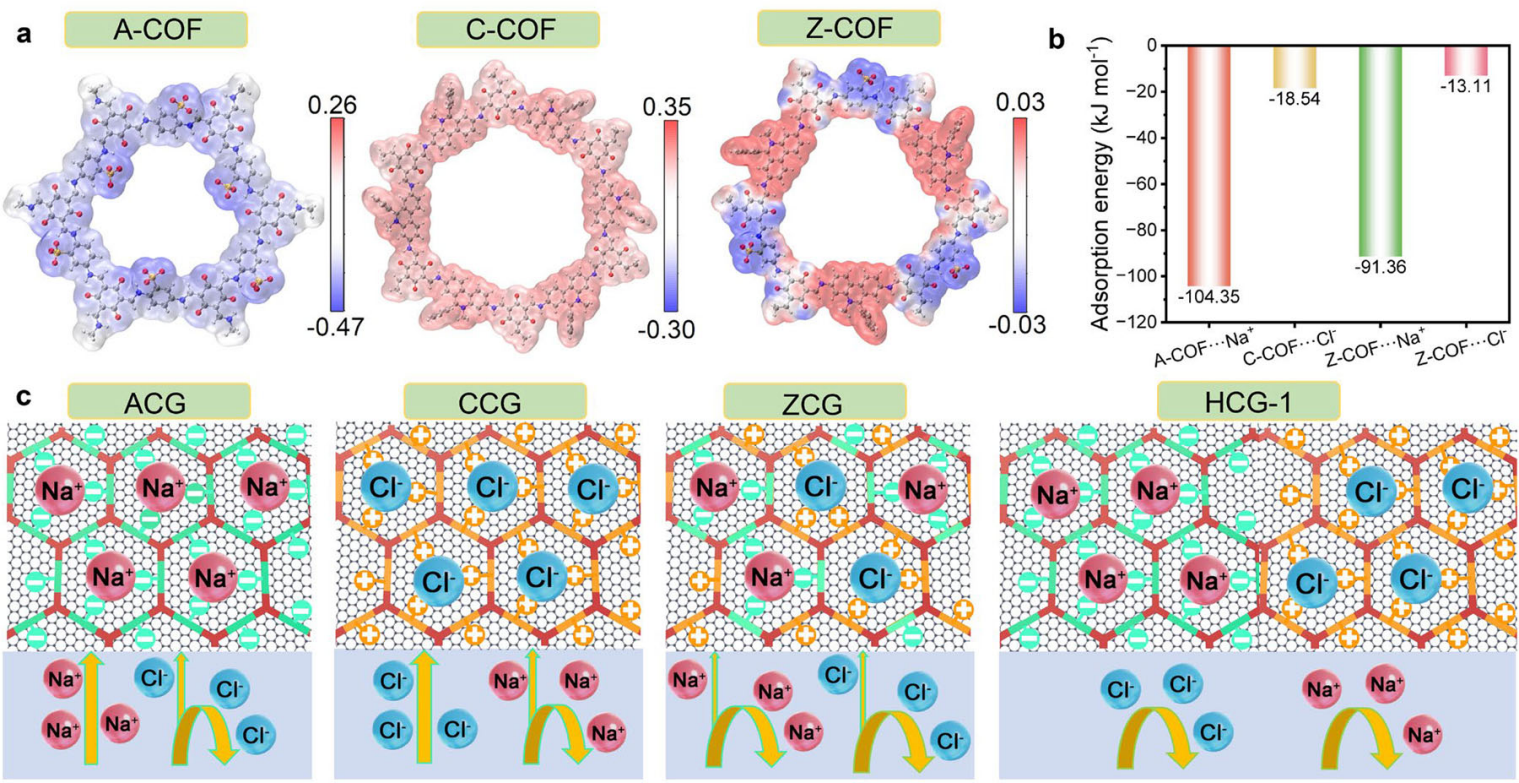
a) ESP distributions of A‐COF, C‐COF and Z‐COF. b) Cl^−^/Na⁺ adsorption energies of A‐COF, C‐COF and Z‐COF. c) Schematics of salt resistance mechanisms of ACG, CCG, ZCG and HCG‐1.

To uncover the origin of the aforementioned differences, the electrostatic potential (ESP) distribution (**Figure** **6**a) and adsorption energies of Cl^−^ and Na^+^ (Figure 6b) in A‐COF, C‐COF and Z‐COF were studied via density functional theory (DFT) calculations.^[^ ^66^, ^67^, ^68^ ^]^ The ESP distribution of the COF frameworks (Figure 6a) reveals distinct charge environments that govern ion adsorption and migration. A‐COF exhibits a predominantly negative potential within its pore channels, strongly favoring Na^+^ adsorption (adsorption energy −104.35 kJ mol^−1^) while interacting only weakly with Cl^−^ (Figure 6c). This asymmetric ion affinity leads to selective Na^+^ enrichment within the hydrogel, a phenomenon reminiscent of the Donnan effect, accompanied by Cl^−^ co‐migration to maintain charge balance. The resulting local ion supersaturation rapidly induces severe salt crystallization on the ACG surface. In contrast, C‐COF displays a positive ESP that strongly binds Cl^−^ (adsorption energy −18.54 kJ mol^−1^), simultaneously attracting Na^+^ to maintain the charge balance. Although this also causes interfacial salt deposition, the overall extent is mitigated by the negatively charged graphene substrate, which weakens local ion accumulation. Z‐COF, with alternating positive and negative domains, shows moderate adsorption towards both Na^+^ and Cl^−^ (adsorption energies −91.36 and −13.11 kJ mol^−1^, respectively). This balanced interaction promotes the migration of hydrated ion pairs rather than free ions, thereby reducing synchronous accumulation at the evaporation interface. However, minor crystallization still occurs owing to the proximity of opposite charges at the molecular scale. In contrast, HCG‐1 integrates oppositely charged COFs into the graphene network, generating spatially separated hetero‐charge domains. These domains act as complementary traps for Na^+^ and Cl^−^, effectively decoupling their co‐migration pathways (Figure 6c). This spatial ion separation prevents localized supersaturation at the evaporation surface and redirects crystallization to the surrounding foam support. Moreover, the hetero‐charge configuration not only stabilizes ion distribution but also enhances water mobility and lowers the enthalpy of vaporization, collectively enabling efficient evaporation and robust salt resistance.

**Figure 7:**
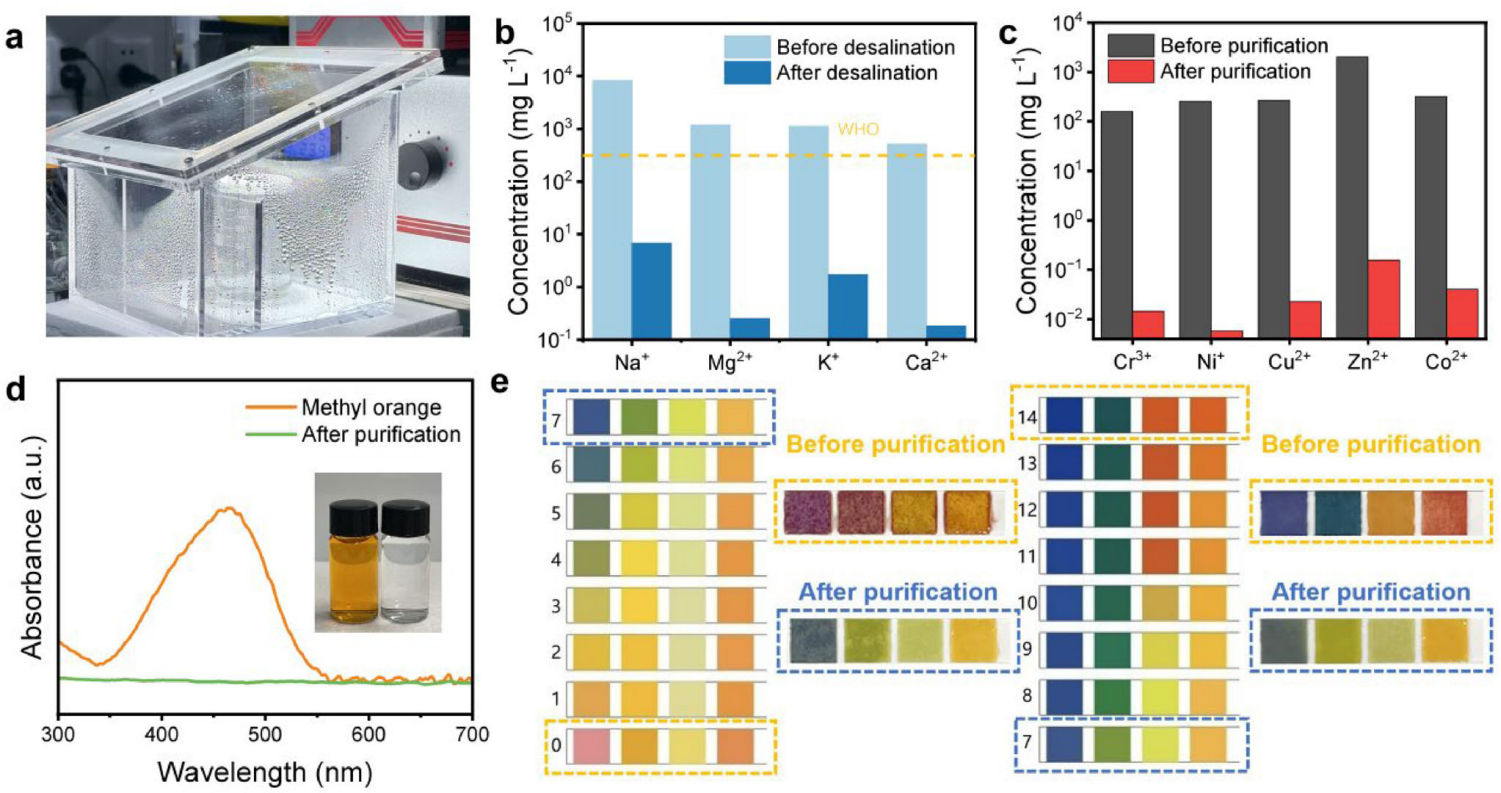
a) Photographs of the water collection device. b) Ion concentrations before and after desalination of seawater. c) Heavy metal ion concentrations before and after purification of seawater. d) Ultraviolet‐visible spectra and photographs of dye‐contaminated and purified water. e) Purification capacity for strong acid and alkali aqueous solution.

To further validate the practical applicability of HCG‐1 in solar‐driven seawater desalination and wastewater treatment, we designed a customized water collection device to collect condensate from seawater and polluted water (**Figure** **7**a). Using real seawater from the Yellow Sea, the treated water quality was analyzed via inductively coupled plasma mass spectrometry (ICP‐MS). After desalination by the solar water evaporation system, the concentrations of four primary ions (Na^+^, Mg^2+^, K^+^ and Ca^2+^) were notably reduced by three orders of magnitude (Figure 7b), meeting the World Health Organization (WHO) standards for drinking water and confirming the system's excellent desalination capability. Additionally, HCG‐1 demonstrated remarkable purification of wastewater containing heavy metal ions (Cr^3+^, Ni^+^, Cu^2+^, Zn^2+^, and Co^2+^), with post‐treatment concentrations markedly reduced (Figure 7c). Furthermore, HCG‐1 was used to treat industrial dye wastewater, with methyl orange and methylene blue as representative pollutants. The results showed that the wastewater was transformed into clear, colorless water following treatment by the solar evaporation system (Figure 7d; Figure S20, Supporting Information). Ultraviolet–visible absorption spectroscopy analysis revealed that the purification efficiency of the collected water was nearly 100%, indicating HCG‐1′s extremely high purification efficiency. Notably, HCG‐1 exhibited excellent purification performance under extreme acidic and alkaline conditions (1 M H_2_SO_4_ and 1 M NaOH). After treatment, the collected water had a pH close to neutral (pH ≈ 7; Figure 7e), further demonstrating the system's chemical robustness and adaptability across diverse water‐treatment scenarios. Collectively, HCG‐1 shows broad application potential in seawater desalination, heavy‐metal wastewater treatment and industrial‐dye wastewater purification, providing a versatile platform for advanced solar‐driven water‐treatment technologies.

## 3 Conclusion

In summary, we successfully develop a series of COF/graphene hydrogels with precisely tunable charge distributions, including hetero‐charged (HCG), zwitterionic (ZCG) and homo‐charged (CCG/ACG) configurations, for efficient solar steam generation. By engineering the charge architecture at the molecular level—particularly by spatially integrating C‐ and A‐COFs on graphene—HCG‐1 achieves a unique evaporation behavior, with a higher rate in saline water than in pure water. This enhanced performance is attributed to the hetero‐charged network, which promotes selective ion–hydrogel interactions, facilitates IW formation, reduces evaporation enthalpy and effectively inhibits salt crystallization at the evaporation interface. In addition, HCG‐1 demonstrates excellent long‐term durability, scale resistance and broad‐spectrum water purification capabilities, including the removal of heavy metals and dyes, as well as efficient water purification under extreme pH conditions. This work not only introduces a new class of tunable‐charge hydrogels for high‐efficiency solar desalination, but also presents an effective design strategy for developing next‐generation solar evaporators with integrated ion‐regulation and anti‐fouling functionality. Moreover, it opens promising avenues for sustainable water treatment in complex, real‐world environments.

## Conflict of Interest

The authors declare no conflict of interest.

## Supporting information



Supporting Information

## Data Availability

The data that support the findings of this study are available from the corresponding author upon reasonable request.
