# Supplementary material for: Engineering Charge Heterogeneity in COF/Graphene Hydrogels for Salt‐Resistant Solar Evaporation
Source: Adv Sci (Weinh). 2025 Dec 2;13(9):e19171. doi: 10.1002/advs.202519171 (PMC12904044; doi:10.1002/advs.202519171)
Supplement: Supplementary file 1 — Supporting Information [file ADVS-13-e19171-s001.pdf]

Supporting Information

Engineering Charge Heterogeneity in COF/Graphene Hydrogels for Salt-Resistant Solar Evaporation

Shuwen Jia[1], Yanrui Li[1], Huangyu He[2], Xinda Nan[1], Xiao Xiao[2], Chuan Wang[1,*], Arne Thomas[3,*], Changxia Li[1,*]

[1] School of Chemistry and Molecular Engineering, Nanjing Tech University, Nanjing 211816, PR China

[2] Department of Chemistry, Westlake University, Hangzhou, Zhejiang 310030, China

[3] Department of Chemistry, Division of Functional Materials, Technische Universität Berlin, Hardenbergstraße 40, 10623 Berlin, Germany

Table of Contents

S1. Supporting Methods

S1.1 Chemicals and materials

S1.2 Fabrication procedures

S1.3 Characterization

S1.4 DFT calculation method

S1.5 Solar vapor generation measurement

S2. Supporting Figures and Tables

S1. Supporting Methods

S1.1 Chemicals and materials

All reagents and solvents are commercially obtained and used as received. *p*-Toluenesulfonic acid (PTSA) was purchased from Sigma-Aldrich. 2,5-Diaminobenzenesulfonic acid (DASA), dimidium bromide (DB), 1,3,5-triformylphloroglucinol (Tp) were purchased from TCI chemicals. Graphite powder (325 mesh) was purchased from Qingdao Huatai Lubricant Sealing S&T Co. Ltd, Qingdao, China.

The seawater in the desalination test came from the Yellow Sea of China and did not require further sterilization. Wastewater with different heavy metal ions of Cr^{3+}, Ni^{2+}, Cu^{2+}, Zn^{2+} and Co^{3+} was prepared by dissolving chromic nitrate, nickel chloride, cupric nitrate, zinc chloride, and cobaltous chloride in deionized water, respectively. MB and MO solutions were prepared by dissolving methylene blue (MB) and methyl orange (MO) in deionized water. The concentrations of MB and MO solution used in the solar purification test were 15 and 20 mg L^{-1}, respectively.

S1.2 Fabrication procedures

S1.2.1 The synthesis of graphene oxide (GO) dispersion

The GO dispersion was prepared from graphite powder using the modified Hummer's method.[1]

S1.2.2 The synthesis of COF

S1.2.2.1 The synthesis of Zwitterionic COF (Z-COF)

0.225 mmol DASA ,0.225 mmol DB and 500 mg PTSA were ground well, then 2 mL of water was added drop by drop. The mixture was ground thoroughly, then transferred into a bottle and 3 mL of water was added. The mixture was shaken well in a vortex shaker for 5 min. Then, 0.3 mmol of Tp was added and the mixture was shaken for another 20 min. The red mixture was transferred into the autoclave with another 5 mL of water and kept in the oven at 120 °C for 48 h. Then, the red precipitate was filtered and sequentially washed with 3M HNO_3 and water. Finally, the collected solid was

Soxhlet extracted with THF and dried overnight at 60 °C.

S1.2.2.2 The synthesis of Anionic COF (A-COF)

0.45 mmol DASA and 500 mg PTSA were dispersed into 4 mL deionized water by ultrasound. 0.3 mmol Tp was then added and shaken for 20 minutes. The mixture was transferred into the autoclave and kept in the oven at 120 °C for 2 days. The product was washed using water for 3 times and subsequently by acetone using Soxhlet extraction, then dried overnight at 60 °C.

S1.2.2.3 The synthesis of Cationic COF (C-COF)

0.45 mmol DB and 500 mg PTSA were ground well, then 1 mL of water was added drop by drop. The mixture was ground thoroughly, then transferred into a bottle and 3 mL of water was added. The mixture was shaken well in a vortex shaker for 5 min. Then, 0.3 mmol of Tp was added and the mixture was shaken for another 20 min. The red mixture was transferred into the autoclave with another 5 mL of water and kept in the oven at 120 °C for 48 h. Then, the red precipitate was filtered and sequentially washed with 3M HNO_3 and water. Finally, the collected solid was Soxhlet extracted with THF and dried overnight at 60 °C.

S1.2.3 The fabrication of COF/rGO hydrogels

S1.2.3.1 The fabrication of HCGs

DASA and PTSA were dispersed into deionized water by ultrasound. 5 mg mL^{-1} GO (1) solution was then added into the solution and shaken for 5 min. Tp was subsequently added and shaken the solution for 20 min to obtain solution 1.

DB and PTSA were ground well, then water was added drop by drop. 5 mg mL^{-1} GO (1) solution was then added into the solution and shaken for 5 min. Tp was subsequently added and shaken the solution for 20 min to obtain solution 2.

After that, the above solutions 1 and 2 were mixed, an extra 5 mg mL^{-1} GO (2) solution was added and the solution was stirred for 30 min, transferred to an autoclave and then kept in the oven at 120°C for 2 days. The resulted hydrogel was washed sequentially

using water, hot acetone and water. The addition amount of each precursor is shown in Table S1.

The hydrogels were also prepared on a large scale using larger autoclaves and all amounts of reactants multiplied times five.

S1.2.3.2 The fabrication of ZCG

DASA, DB and PTSA were ground well, then water was added drop by drop. 5 mg mL^{-1} GO (1) solution was then added into the solution and shaken for 5 min. Tp was subsequently added and shaken the solution for 20 min. After that, an extra 5 mg mL^{-1} GO (2) solution was added and the solution was stirred for 30 min, transferred to an autoclave and then kept in the oven at 120 °C for 2 days. The resulted hydrogel was washed sequentially using water, hot acetone and water. The addition amount of each precursor is shown in Table S1.

S1.2.3.3 The fabrication of ACG and CCG

S1.2.3.3.1 The fabrication of ACG

DASA and PTSA were dispersed into deionized water by ultrasound. 5 mg mL^{-1} GO (1) solution was then added into the solution and shaken for 5 min. Tp was subsequently added and shaken the solution for 20 min. After that, an extra 5 mg mL^{-1} GO (2) solution was added and the solution was stirred for 30 minutes, transferred to an autoclave and then kept in the oven at 120 °C for 2 days. The resulted hydrogel was washed sequentially using water, hot acetone and water. The addition amount of each precursor is shown in Table S1.

S1.2.3.3.2 The fabrication of CCG

DB and PTSA were ground well, then water was added drop by drop. 5 mg mL^{-1} GO (1) solution was then added into the solution and shaken for 5 min. Tp was subsequently added and shaken the solution for 20 min. After that, an extra 5 mg mL^{-1} GO (2) solution was added and the solution was stirred for 30 min, transferred to an autoclave and then kept in the oven at 120 °C for 2 days. The resulted hydrogel was washed sequentially

using water, hot acetone and water. The addition amount of each precursor is shown in Table S1.

S1.3 Characterization

Fourier transform infrared spectroscopy (FT-IR) analyses were conducted on a Bruker VERTEX 70v FT-IR Spectrometer with Golden Gate ATR module from Specac. Nitrogen physisorption isotherms were measured at -196°C using a Micromeritics 3FLEX instrument. The sample was degassed under vacuum at 120 °C for 12 h before the measurement. The surface area was calculated using the Brunauer-Emmett-Teller (BET) method and pore size distribution was calculated from the adsorption branch of isotherms using the non-localized density functional theory (NLDFT) model. The X-ray photoelectron spectroscopy (XPS) analysis was performed using a Thermo Fisher ESCALAB Xi+ instrument with Al Kα radiation, The samples were mounted on the holder using carbon tape. The powder X-ray diffraction (XRD) was performed on a Bruker D8 Advance diffractometer equipped with a LynxEye XE-T detector, with a 2θ step size of 0.02° and a scanning range of 2–50°. Differential scanning calorimetry (DSC) analyses were performed on a Netzsch instrument (STA 449-F3 Jupiter) under an N_2 flow of 50 mL min^{-1} with a heating rate of 5 °C min^{-1}, from 25 to 180 °C. The Scanning electron microscopy (SEM) analysis was performed on a KYKY-EM8100 electron microscope. Transmission electron microscopy (TEM) analysis was conducted on a FEI Talos L120C electron microscope. Energy-dispersive X-ray spectroscopy (EDS) was performed on a Thermo Fisher Talos F200X G2 instrument. Atomic force microscopy (AFM) was tested using a Bruker Dimension ICON model. Freshly prepared samples were spin coated (800 rpm, 100 seconds) on mica sheets after ultrasonication for 30 minutes. Raman spectra were characterized on WITec alpha 300A with an exciting laser wavenumber of 532 nm. Optical contact angle measurements and analysis were performed at room temperature with OCA 15+ device with LDU and SCA20 software, dataphysics GmbH. UV-Vis-NIR spectrometer (Cary 5000) measured the absorption spectrum. The spectrometer is equipped with an integrating sphere device and an automatic reflection measurement device. Solar evaporator tests were

performed using a solar simulator (CEL-PE300L-3A) outputting stable solar flux at 1000 W m^{-2} (1 sun) with collimated beam with 40 mm diameter. The light intensity was always calibrated before tests. Zeta Potential Analyzer (BI-200SM/NanoBrook ZetaPALS/BI-DNDC) was used to test the surface potential of samples with a zeta potential range of -500mV to 500mV. The concentration of heavy metal ions in solution was measured using a Shimadzu ICPMS-2030 Inductively Coupled Plasma Mass Spectrometer (ICP-MS). The concentration of dye in solution was measured using a Mettler-Toledo UV5Bio UV-Vis spectrophotometer.

S1.4 DFT calculation method

Molecular dynamics simulations employing xtb[2] at the GFN0-xTB level were carried out to explore the conformations of Cationic COF, Anionic COF and Zwitterionic COF. The resulting structures are processed using Molclus[3] to identify and select the frequently occurring conformation as the subject of further study. These representative COF structures, and their Na^+ or Cl^- adsorption complexes were then optimized using Gaussian 16 program package[4] at the ωB97XD/6-31G** level[5-7] with IEFPCM water solvation model to account for aqueous solvation effects.[8-10] Single-point energies calculations were then performed at the ωB97XD/6-311+G**level with the SMD water solvation model.[11] The electrostatic potential was generated with Multiwfn,[12-13] and visualized in VMD (version 1.9.3).[14] Adsorption energies were calculated according to the equation:

$$E_{adsorption} = E_{complex} - E_{COF} - E_{ion}$$

where $E_{complex}$, E_{COF} and E_{ion} denote the electronic energies of the optimized COF-ion complex, isolated COF, and isolated ion, respectively.

S1.5 Solar vapor generation measurement

S1.5.1 Water vapor generation experiments

The circular CGs (2.1–2.5 cm^2 area, 2 mm thickness) were placed at the center of an expandable polystyrene (EPS) foam so that the bottom was in direct contact with the water below. The samples were irradiated by a solar simulator under stable solar irradiation. The surface temperature changes of the samples were recorded using an infrared thermometer. Under steady-state conditions, the evaporation rate of the samples was measured. Before testing, a digital caliper was used to measure the diameter of each sample to calculate the actual surface area. All hydrogel evaporators used for evaporation measurements were carefully controlled to a consistent effective evaporation area of 2.1–2.5 cm^2, ensuring full comparability across all samples. The weight loss of the entire setup was recorded using an electronic balance. The dark evaporation process was measured for 10 h in a dark environment under ambient conditions to record the weight loss. Stability experiments were conducted for 12 consecutive days with a daily test duration of 10 hours. At the end of each day's testing, the samples were left untreated and testing continued the following day to assess their stability under prolonged continuous use conditions.

S1.5.2 Water vapor collection experiment

The above device was put into a home-made glass container. The sloping lid of this container permits solar light to completely transmit. The formed water steam then condensates on the glass wall.

S1.6 Statistical analysis

The data processing was conducted using Origin Pro software (OriginLab Corp.). The data about water evaporation results, zeta potential results, and DSC results are presented as the average and standard deviation of three tests per sample.

S2. Supporting Figures and Tables

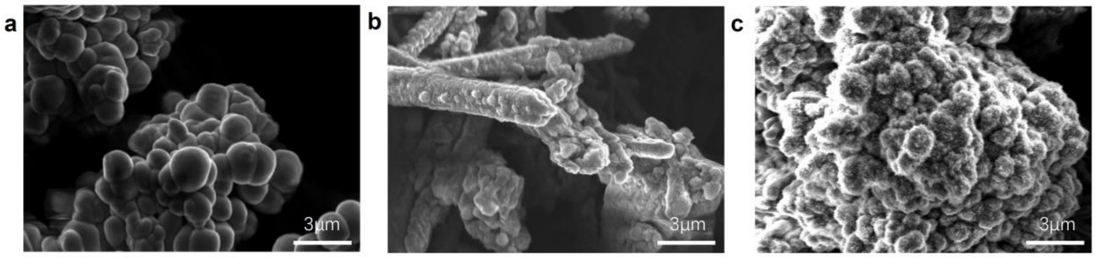

Figure S1. SEM images of (a) Z-COF, (b) C-COF and (c) A-COF.

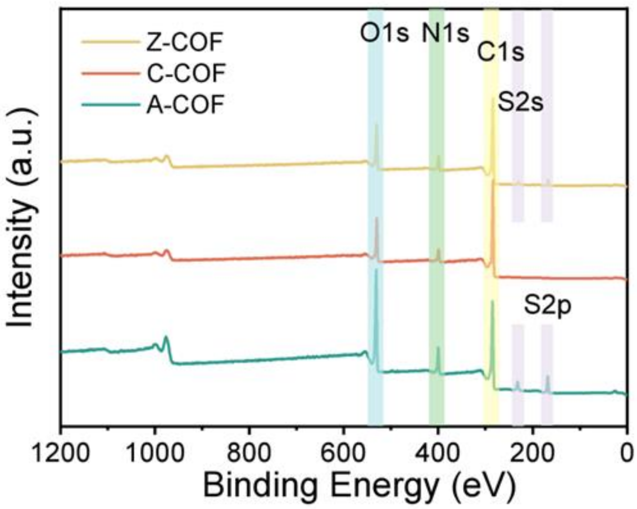

Figure S2. XPS survey spectra of Z-COF, C-COF and A-COF.

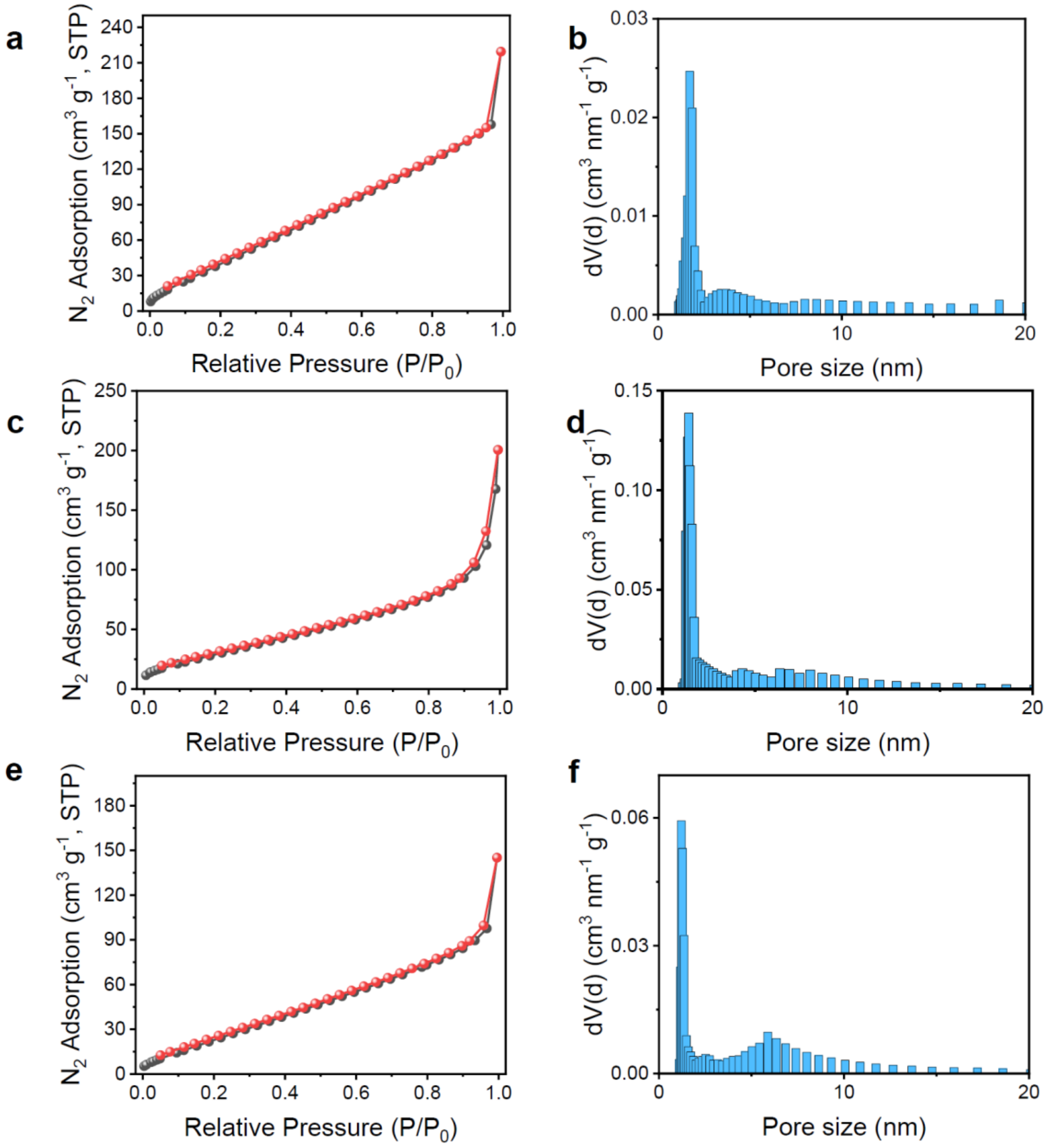

Figure S3. N_2 adsorption-desorption isotherms and pore size distributions of (a, b) Z-COF, (c, d) A-COF, and (e, f) C-COF.

Table S1. Amount of reactants used for preparing the hydrogels. The concentration of GO dispersion used in all experiments is 5.0 mg mL^{-1}.

Sampels	PTSA	DASA	DB	H_2O	GO(1)	TP	GO(2)
HCG-1	33.06mg	5.64mg		1ml	2.5ml	4.2mg	2.5ml
	33.06mg		11.4mg	1ml	2.5ml	4.2mg	
HCG-2	22.04mg	3.76mg		1ml	2.5ml	2.8mg	2.5ml
	44.08mg		15.2mg	1ml	2.5ml	5.6mg	
HCG-3	44.08mg	7.52mg		1ml	2.5ml	5.6mg	2.5ml
	22.04mg		7.6mg	1ml	2.5ml	2.8mg	
ZCG	66.12mg	5.64mg	11.4mg	2ml	5ml	8.4mg	2.5ml
ACG	82.65mg	14.10mg		2ml	5ml	10.5mg	2.5ml
CCG	55.10mg		19mg	2.5ml	5ml	7.0mg	5ml

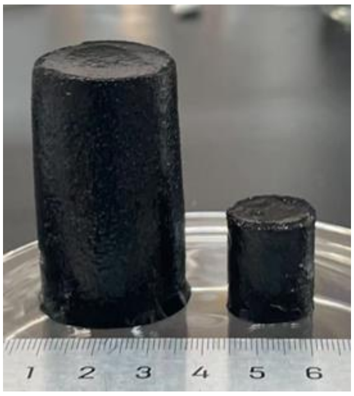

Figure S4. Photograph of HCG-1 synthesized using 100 mL (left) and 20 mL (right) autoclaves.

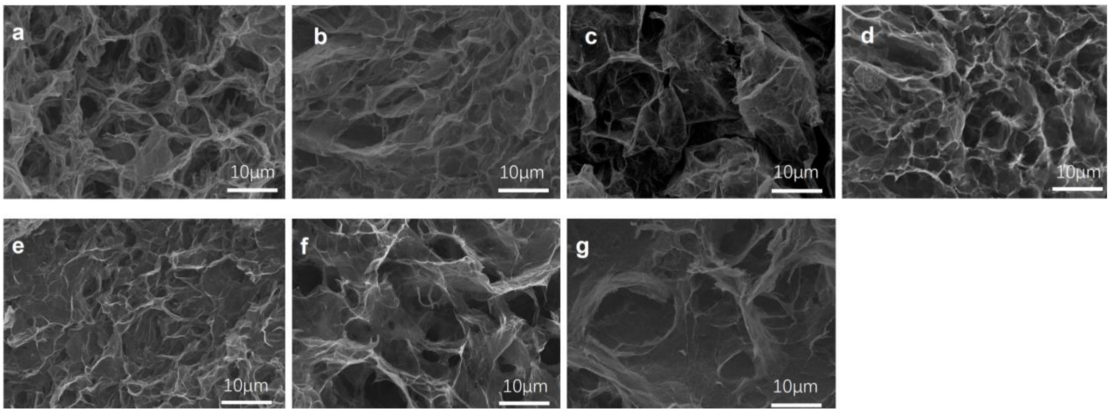

Figure S5. SEM images of (a) HCG-1, (b) HCG-2, (c) HCG-3, (d) ZCG, (e) ACG, (f) CCG and (g) rGO.

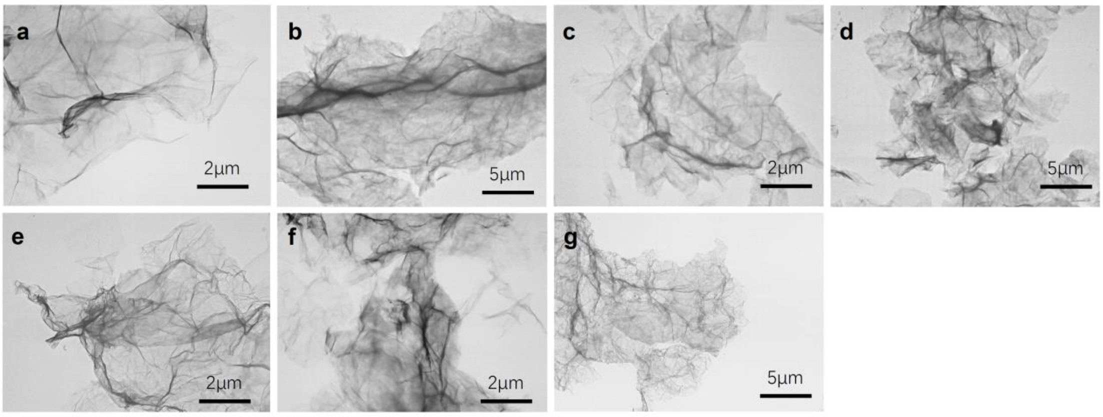

Figure S6. TEM images of (a) HCG-1, (b) HCG-2, (c) HCG-3, (d) ZCG, (e) ACG, (f) CCG and (g) rGO.

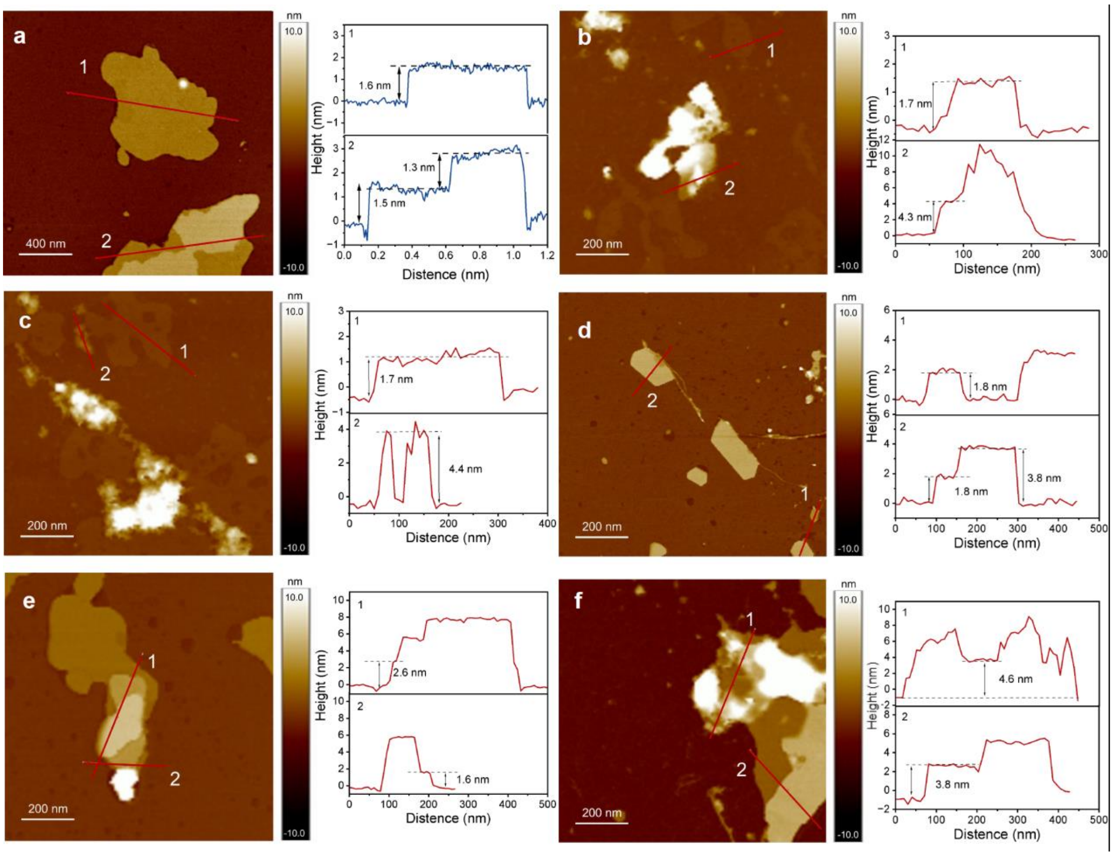

Figure S7. AFM images and the corresponding height profiles for (a) rGO, (b) HCG-2, (c) HCG-3, (d) ZCG, (e) ACG, (f) CCG.

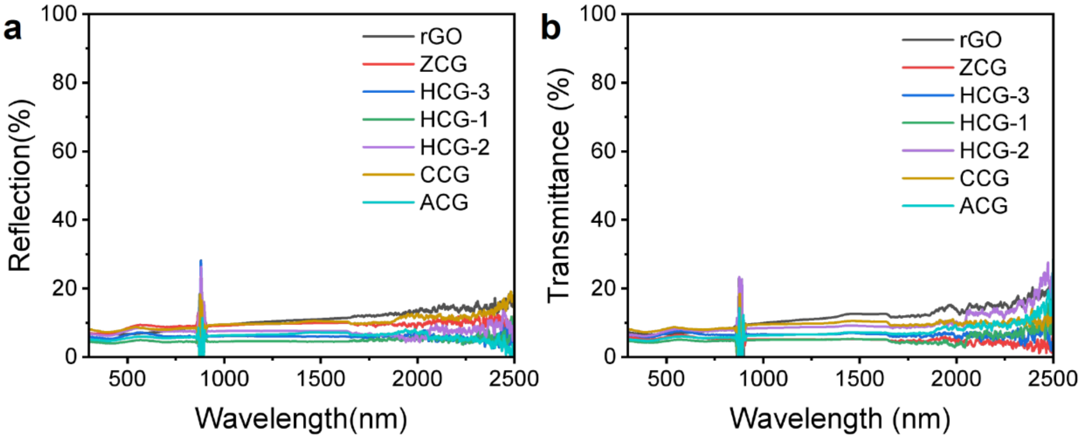

Figure S8. (a) Reflectance spectra of CGs in the wavelength range of 250-2500 nm. (b) Transmittance spectra of CGs in the wavelength range of 250-2500 nm.

The absorbance (*A*), reflectance (*R*), and transmittance (*T*) of materials satisfy the following formula:

$A+R+T=1$

To quantify the absorption of CGs, the reflectance and transmittance of CGs were tested using UV-Vis NIR in the spectral range of 250-2500 nm. Thus, the absorbance of CGs in the range of 250-2500 nm was calculated.

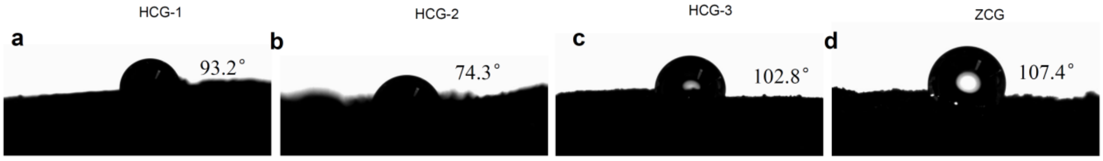

Figure S9. Water contact angle of the lyophilized samples.

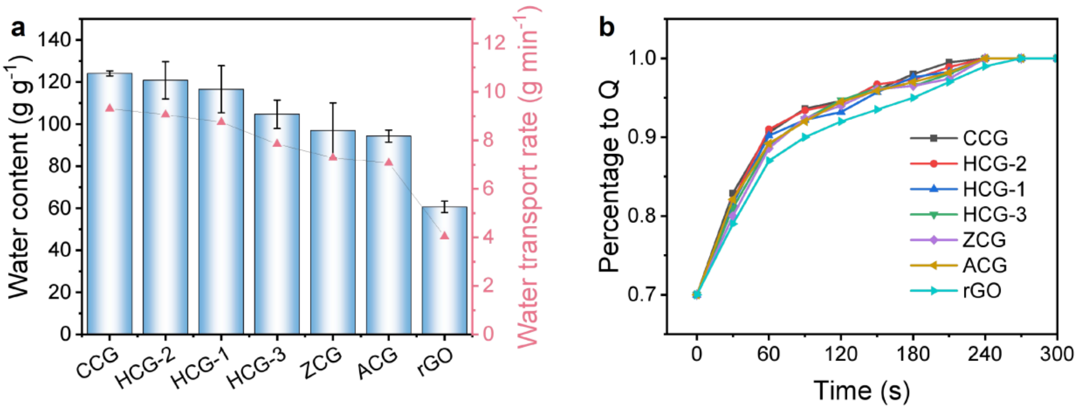

Figure S10. (a) Water content of CGs, and the water transport rate calculated from the swelling behavior from semi-saturated to saturated states shows the adjustable water transport capacity of CGs. (b) Percentage to the corresponding saturated water content (Q) over time of each sample.

Considering that the CGs are nearly completely hydrated during the evaporation, we use absorbent paper to partially remove water from the capillary channels of saturated hydrogel and carefully control the water content in the hydrogel down to 70% of the original value (i.e., 0.7Q). Figure S10 (b) shows the corresponding percentage to saturated water content (Q) over time. The water transport rate (V) can be calculated by

$$V = (1\text{-}0.7)Q/t$$

where t is the water uptake time from 0.7Q to the saturated state (Q). The V values from CCG to rGO are 9.31, 9.06, 8.75, 7.85, 7.28, 7.07 and 4.04 g min^{-1}, respectively, revealing the water transport behavior in the hydrogels.

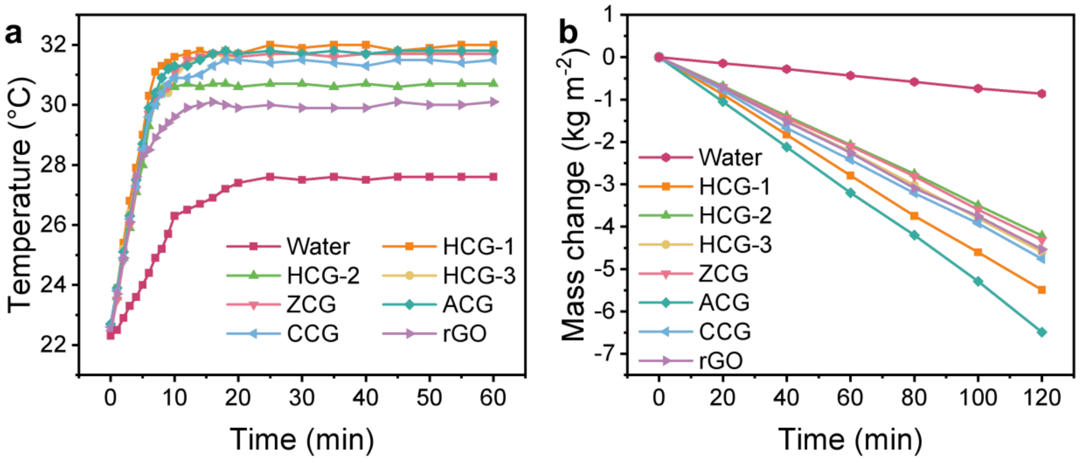

Figure S11. Time-dependent (a) temperature and (b) mass change of pure water and CGs in pure water under 1 sun irradiation (1 kW m^{-2}).

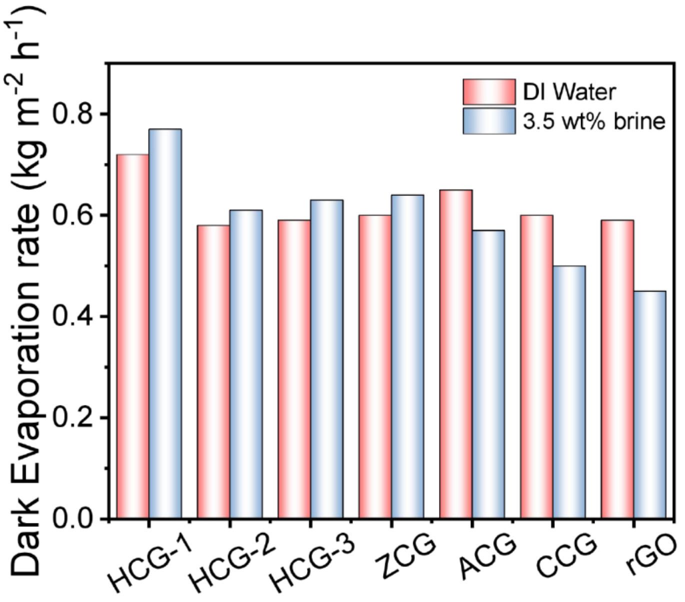

Figure S12. Dark evaporation rates for all samples in water and 3.5 wt% brine.

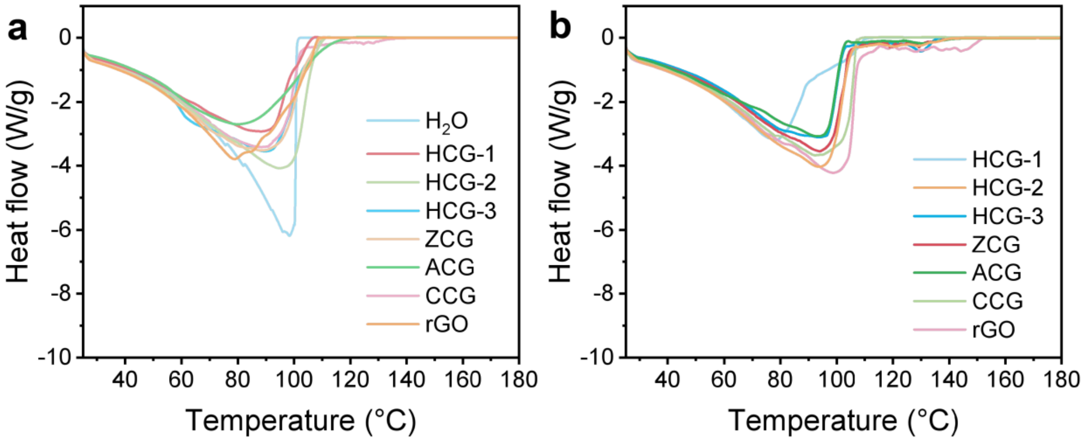

Figure S13. (a) Thermograms of pure water and CGs in pure water. (b) Thermograms of CGs in 3.5 wt% brine.

From the heat flow signal, a sharp peak can be observed for pure water. The signal drops sharply after it reaches its maximum value, which indicates that the evaporation of water is completed immediately. For all CGs, the peak is broader than for pure water, and the heat flow shows a gradual decay, indicating that the evaporation of water in CGs is different from that of pure water.

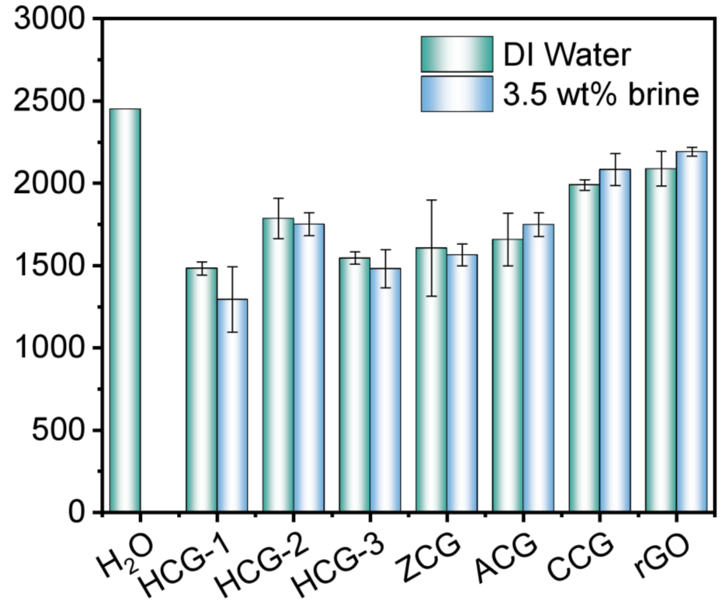

Figure S14. The enthalpy of evaporation of water in CGs under dark conditions.

Estimation of energy consumption by dark experiment

According to the literature method,[15] the evaporation enthalpy of hydrogels could be estimated using a designed experiment. Pure water and hydrogel samples with same superficial area were synchronously located in a closed container with supersaturated potassium carbonate solution to enable stabilized relative humidity of ~45% at room temperature and ambient air pressure. Their mass changes under dark condition were recorded to estimate the evaporation enthalpy of water in hydrogels using the following equation:

$U_{in}=E_{equ}m_h=E_0m_0$

where E_0 is the evaporation enthalpy of water, m_0 is mass change of pure

water (without hydrogel evaporator) under the dark condition and m_h is the mass change of the hydrogels under the same conditions. The evaporation enthalpy value (E_{equ}) could be calculated by making a comparison with the known theoretical value of liquid water (2450 J/g) using unchanged power input (U_{in}).

The dark environment evaporation test is simple and close to the actual evaporation process, which is suitable for evaluating the evaporation performance of materials under real conditions, and the evaporation process only evaporates free water and intermediate water, and thus the relative value is lower than that of the DSC test. In contrast, the DSC test can evaporate various types of water, including bound water, intermediate water and free water, making the samples completely dewatered. However, the test conditions differ greatly from the actual evaporation process and cannot reflect the photothermal effect under light. Both of them have their own focuses, and can be used together to evaluate the evaporation behavior of materials more comprehensively.

Variation of water state in CGs

In the hydrogel network, COF is super-hydrophilic, thus can attract large amount of water molecules.[16-17] There are three kind of water states in a hydrogel: bound water (BW), intermediate water (IW) and free water (FW).[18-19] BW strongly interacts with hydrophilic groups and does not participate in water evaporation. The rate of water evaporation is determined by the IW and FW. Free water, also called bulk water, has no interaction with groups in hydrogels. IW is a state between BW and FW with weak interaction with surrounding water molecules. In CGs, the hydrogels provide multiple $-SO_3^-$ and $-NR_4^+$ groups that form hydrogen bonds with water molecules, thereby weakening the hydrogen bonding network among adjacent water molecules. These surrounding water molecules are IW. Therefore, increasing the IW content can lower energy barrier and accelerate water evaporation. The different water states in CGs were investigated by Raman spectroscopy. The Raman spectra were fitted through Gaussian functions. The three peaks at low wavenumber represent vibration of chemical bonds (2600–3000 cm^{-1}) and Fermi resonance (3060 cm^{-1}), respectively. The peaks observed at 3200, 3350, 3450, and 3610 cm^{-1} are related to water. The four peaks are classified as two types of modes: (1) the peaks at 3200 and 3350 cm^{-1} are assigned to FW, corresponding to tetrahedral H-bonding, i.e., a water molecule forming four H bonds with surrounding water molecules; (2) the peaks at 3450 and 3610 cm^{-1} are

corresponding to IW with fewer or broken hydrogel bonds, which weakly interact with adjacent water molecules.

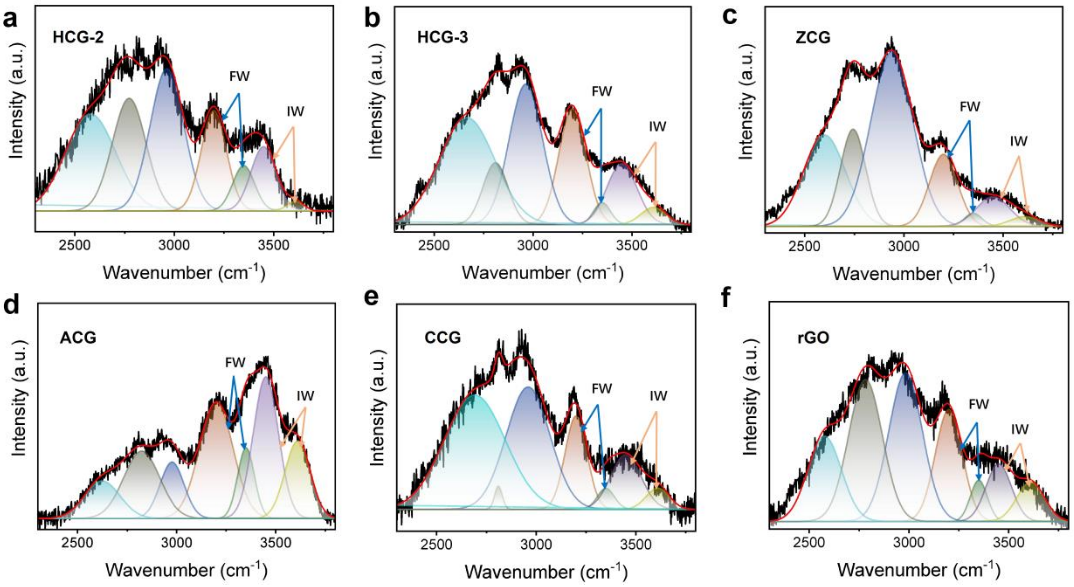

Figure S15. Raman spectra with fitting curves showing of FW and IW of HCG-2, HCG-3, ZCG, ACG, CCG and rGO in pure water.

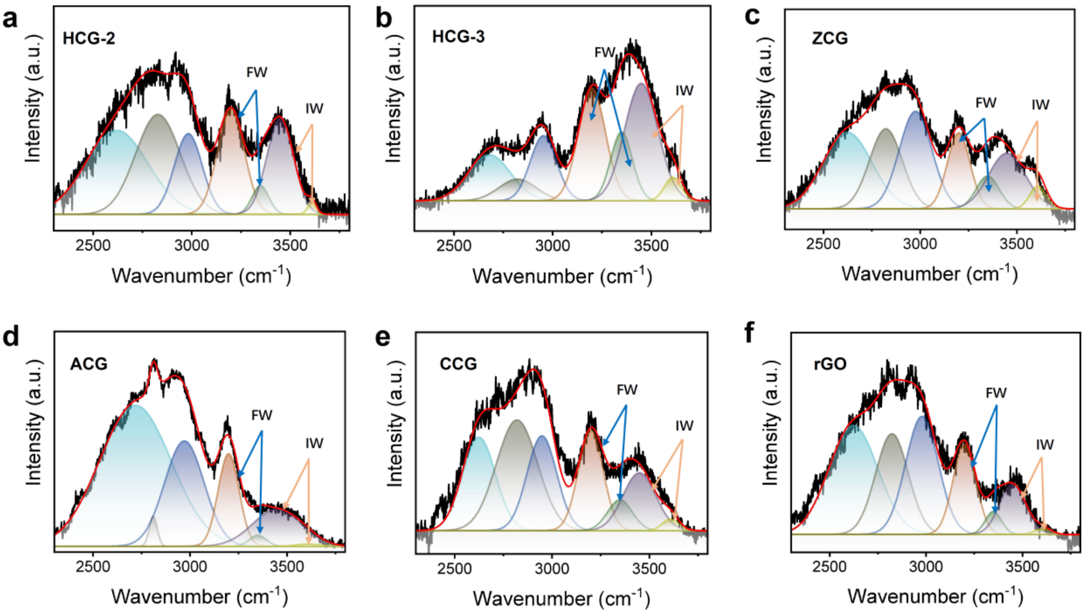

Figure S16. Raman spectra with fitting curves showing of FW and IW of HCG-2, HCG-3, ZCG, ACG, CCG and rGO in 3.5 wt% brine.

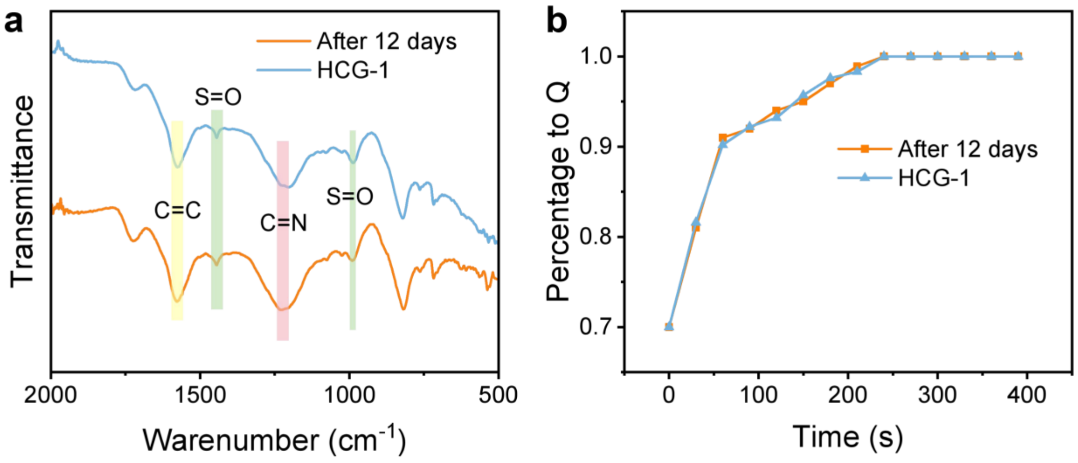

Figure S17. (a) FT-IR spectra of HCG-1 and HCG-1 after 12 days stability test. (b) Percentage to the corresponding saturated water content (*Q*) over time of HCG-1 and HCG-1 after 12 days stability test.

The *V* values are 8.75 g min^{-1} and there was little difference between the before and after stability test samples.

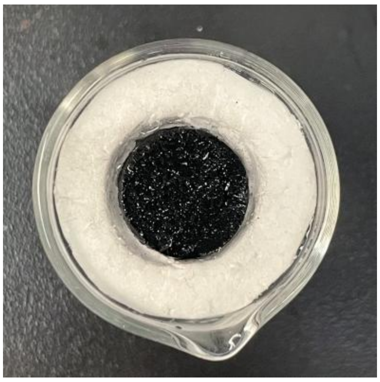

Figure S18. The hydrogel was subjected to 7 h of continuous light exposure under one sun using seawater, and no salt crystallized on the surface.

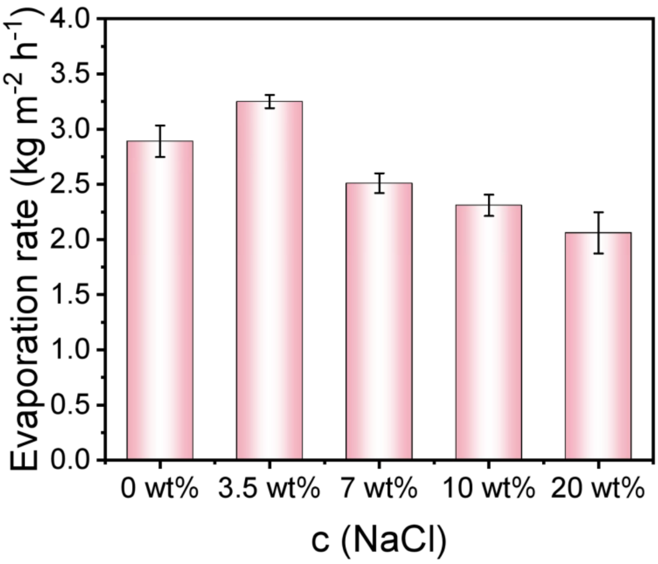

Figure S19. Evaporation rate of HCG-1 in different brines.

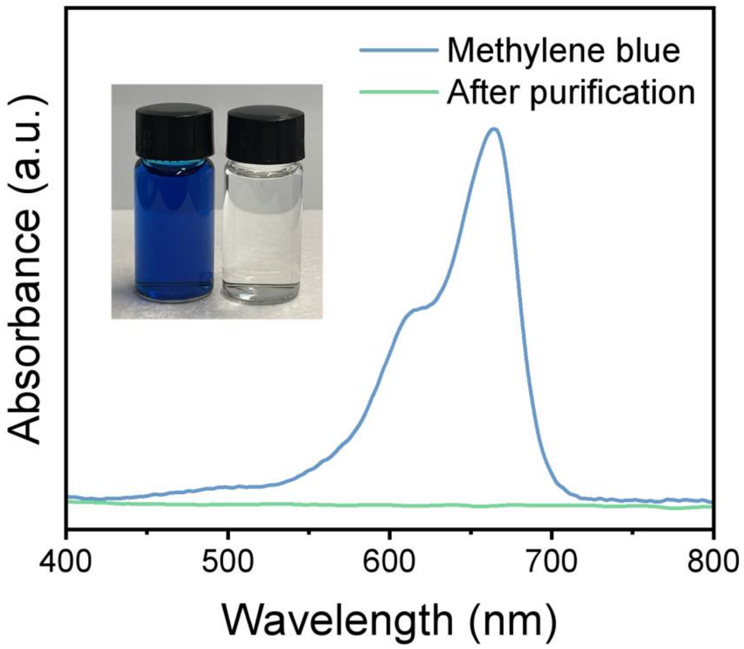

Figure S20. UV−vis spectra and photograph of MB dye contaminated water and purified water.

Table S2. Content (at%) of Br, S, N, C and O elements for all the samples as measured by XPS.

Sampels	Br	S	N	C	O
Z-COF	0.1	2.93	10.03	53.5	33.44
A-COF	0	5.49	10.05	47.18	37.28
C-COF	0.06	0	7.64	61.47	30.83

References

[1] Y. Xu, K. Sheng, C. Li, G. Shi, *ACS Nano* **2010**, *4*, 4324-4330.

[2] C. Bannwarth, E. Caldeweyher, S. Ehlert, A. Hansen, P. Pracht, J. Seibert, S. Spicher, S. Grimme, *WIREs Computational Molecular Science* **2020**, *11*, e1493.

[3] L. Tian., Molclus program, Version 1.12, http://www.keinsci.com/research/molclus.html (accessed Dec. 14th, 2023).

[4] H. Yang, Z. Hu, Z. Huang, S. Wu, J. Yan, K. Cen, Z. Bo, G. Xiong, K. Ostrikov, *Nano Energy* **2024**, *127*, 109784.

[5] J.-D. Chai, M. Head-Gordon, *Physical Chemistry Chemical Physics* **2008**, *10*, 6615-6620.

[6] D. Feller, *Journal of Computational Chemistry* **1996**, *17*, 1571-1586.

[7] K. L. Schuchardt, B. T. Didier, T. Elsethagen, L. Sun, V. Gurumoorthi, J. Chase, J. Li, T. L. Windus, *Journal of Chemical Information and Modeling* **2007**, *47*, 1045-1052.

[8] E. Cancès, B. Mennucci, J. Tomasi, *The Journal of Chemical Physics* **1997**, *107*, 3032-3041.

[9] B. Mennucci, J. Tomasi, *The Journal of Chemical Physics* **1997**, *106*, 5151-5158.

[10] B. Mennucci, E. Cancès, J. Tomasi, *The Journal of Physical Chemistry B* **1997**, *101*, 10506-10517.

[11] C. J. C. Aleksandr V. Marenich, and Donald G. Truhlar, *The Journal of Physical Chemistry B* **2009**, *113*, 6378-6396.

[12] T. Lu, F. W. Chen, *JOURNAL OF COMPUTATIONAL CHEMISTRY* **2012**, *33*, 580-592.

[13] J. Zhang, T. Lu, *Physical Chemistry Chemical Physics* **2021**, *23*, 20323-20328.

[14] W. Humphrey, A. Dalke, K. Schulten, *JOURNAL OF MOLECULAR GRAPHICS & MODELLING* **1996**, *14*, 33-38.

[15] Y. Guo, H. Lu, F. Zhao, X. Zhou, W. Shi, G. Yu, *Advanced Materials* **2020**, *32*, 1907061.

[16] C. Li, S. Cao, J. Lutzki, J. Yang, T. Konegger, F. Kleitz, A. Thomas, *Journal of the American Chemical Society* **2022**, *144*, 3083-3090.

[17] C. Li, J. Florek, P. Guggenberger, F. Kleitz, *Journal of Materials Chemistry A* **2025**, *13*, 214-219.

[18] F. Zhao, X. Zhou, Y. Shi, X. Qian, M. Alexander, X. Zhao, S. Mendez, R. Yang, L. Qu, G. Yu, *Nature Nanotechnology* **2018**, *13*, 489-495.

[19] X. Zhou, Y. Guo, F. Zhao, W. Shi, G. Yu, *Advanced Materials* **2020**, *32*, 2007012.
